# Comparative genomics and synteny analysis of PP2C phosphatases in modern and wild sugarcane cultivars for insights into abiotic stress response

**DOI:** 10.3389/fpls.2025.1596800

**Published:** 2025-08-19

**Authors:** Shweta Kumari, R. K. Harshavardhini, Nandhini Murugan, S. Keerthana, Aispriya, Vinayaka, Anitha Ramaswamy, Jeyakumar Prabhakaran, Manimekalai Ramaswamy

**Affiliations:** ^1^ Division of Crop Improvement, ICAR- Sugarcane Breeding Institute, Coimbatore, Tamil Nadu, India; ^2^ Department of Computer Science, Amrita Vishwa Vidyapeetham, Coimbatore, Tamil Nadu, India; ^3^ Statistics & Economics Section, ICAR-Sugarcane Breeding Institute, Coimbatore, Tamil Nadu, India; ^4^ Department of Crop Physiology, Tamil Nadu Agricultural University, Coimbatore, Tamil Nadu, India

**Keywords:** PP2C phosphatases, evolution, conserved, synteny, stress response

## Abstract

PP2C phosphatases regulate key physiological processes in plants, essential for growth, development, and stress responses. Sugarcane, a vital crop for many economies, faces severe abiotic stress, which negatively impacts production. Given the role of the PP2C gene family in stress tolerance and the recent publication of the genome sequence of the modern polyploid sugarcane cultivar R570, this study conducted genome-wide identification and characterization of the PP2C gene family in sugarcane. The analysis includes genome-wide identification, phylogenetic analysis, gene structure, conserved motif and domain analysis, synteny analysis, evolutionary selection pressure (Ka/Ks) estimation, gene ontology annotation, and RT-qPCR based expression profiling of selected PP2C genes. A total of 500 PP2C genes were identified, distributed across all 10 chromosomes and their subgenomes. Phylogenetic analysis, using Arabidopsis, *S. spontaneum*, maize, and wheat as references, classified these genes into 13 subfamilies. The results showed that, similar to *S. spontaneum*, where the largest subfamily is F with 37 members, the largest subfamily in the sugarcane hybrid cultivar was also F, with 74 members, followed by subfamily A with 69 members. The exon and motif distribution were found to be highly conserved within the same subfamily. Tandem duplication was prominent, with 107 genes identified as paralogs, indicating their role in gene expansion. The chromosomal distribution of SoffiXPP2C genes was partially biased, as approximately 50% of the genes were located on chromosomes 1, 2, and 3, along with their respective subgenomes. Synteny analysis revealed a strong conservation of the protein phosphatase domain between modern hybrid and wild sugarcane (*S. spontaneum*). Additionally, the synteny association of SoffiXPP2C genes with two potential cold stress-responsive genes, SsPP2C27 and SsPP2C64, suggests a possible role of SoffiXPP2C genes in abiotic stress regulation. The observed downregulation of certain SoffiXPP2C genes in waterlogging-tolerant genotypes further supports their potential function as negative regulators, particularly under waterlogging stress conditions. The domain architecture analysis further emphasizes the multifaceted roles of sugarcane PP2Cs, particularly in stress signaling, protein phosphorylation regulation, and membrane-associated functions. Furthermore, the strong alignment of 34 SoffiXPP2C genes with differentially expressed contigs of sugarcane under oxidative stress conditions reinforces insights into the potential role of SoffiXPP2Cs in stress response. These findings provide valuable insights into the evolutionary conservation of PP2C genes in sugarcane hybrid cultivar and their critical role in abiotic stress responses.

## Introduction

Protein phosphorylation and dephosphorylation are vital regulatory mechanisms in plants, orchestrated by protein kinases (PKs) and protein phosphatases (PPs). These processes influence a wide array of physiological functions, including growth, development, metabolism, and responses to environmental stimuli (Xin et al., 2024; Sojka et al., 2024). PKs transfer phosphate groups from ATP to specific amino acids, primarily serine, threonine, or tyrosine on target protein, while PPs remove these phosphate groups, thus modulating protein activity and function. PPs are classified into three main types based on their substrate specificity: serine/threonine protein phosphatases (STPs), tyrosine protein phosphatases (PTPs), and dual-specificity protein phosphatases (DSPTPs) (Saini et al., 2023; Xin et al., 2024). Further classification within the STPs includes the phosphoprotein phosphatase (PPP) family, which is subdivided into PP2A, PP2B, and PP2C based on substrate specificity and sensitivity to inhibitors (Luan, 2000; Cohen, 1997; Xin et al., 2024). The PP2C family is the largest group of protein phosphatases in plants, constituting 60–65% of all phosphorylases. These phosphatases play key regulatory roles in various biological processes, including ABA signaling, biotic and abiotic stress responses, plant immunity, K+ nutrient signaling, and plant development (Kerk et al., 2008; Singh et al., 2010). PP2C proteins are essential regulators across various organisms, from prokaryotes to eukaryotes, including plants, due to their conserved structure and function (Qiu et al., 2022). A common feature of all PP2C-type phosphatases is the presence of 11 characteristic subdomains in the catalytic part of the protein. Their enzymatic activity is dependent on divalent metal ions such as Mn²^+^ or Mg²^+^. In eukaryotic PP2Cs, the catalytic domain may be located either at the N-terminus or C-terminus of the protein (Shi, 2009; Schweighofer et al., 2004; Bheri et al., 2021).

Previous studies have identified 78, 80, 103, 131, 145, and 257 PP2C candidate genes in rice (*Oryza sativa*), Arabidopsis (*Arabidopsis thaliana*), maize (*Zea mays*), mustard (*Brassica rapa*), wild sugarcane (*Saccharum spontaneum*), and wheat (*Triticum aestivum*), respectively (Xue et al., 2008; Wu H. et al., 2023; Khan et al., 2019; Huang et al., 2023; Yu et al., 2019). Based on phylogenetic analysis, the PP2C gene family in Arabidopsis is classified into 10 subgroups (A–J), with group A containing most of the PP2C genes involved in abscisic acid (ABA) signaling (Schweighofer et al., 2004). Several PP2C genes have been reported to play key regulatory roles in abiotic stress responses. In maize, ZmPP2C15, a PP2C family gene, positively regulates drought tolerance by enhancing antioxidant enzyme activity and osmoregulatory substance content, making it a promising target for breeding drought-resistant varieties (Pang et al., 2024). In rice, OsPP65, a PP2C gene, negatively regulates osmotic and salt stress responses by influencing jasmonic acid (JA) and abscisic acid (ABA) biosynthesis, as well as raffinose family oligosaccharide metabolic pathways (Liu et al., 2022). The overexpression of the PP2C gene GhDRP1 in cotton has been shown to reduce drought tolerance, while silencing this gene enhances drought resistance. This phenomenon is attributed to the modulation of stress-related gene expression and flavonoid biosynthesis pathways under drought conditions (Chen et al., 2021). Kołton et al. (2025) showed tissue specific responses, with decreased PP2A-C in leaves but a six-fold increase and doubled activity in roots of tomato plants under water logging stress. Numerous studies highlight the crucial roles of PP2C family genes in regulating abiotic stresses like temperature extremes, drought, and hormonal responses, emphasizing their importance in plant stress adaptation and resilience.

Sugarcane, a key agricultural crop, plays a crucial role in global economies and trade, contributing 80% of the world’s sugar production (Vinayaka and Prasad, 2024). Modern sugarcane cultivars are hybrid cultivars with highly polyploid and enormous genomes (approximately 10 gigabases, Gb). They originate from the interspecific hybridization of the cultivated species *Saccharum officinarum* and the wild species *Saccharum spontaneum* (Surya Krishna et al., 2023; Healey et al., 2024). Despite its significance, sugarcane faces major challenges from abiotic stress, which negatively impacts production. Sugarcane suffers significant yield losses ranging from 30% to 50% due to abiotic stresses, primarily water deficit and heat stress (Kasirajan et al., 2018; Misra et al., 2022). Enhancing stress tolerance in cultivars is crucial for improving yield (Meena et al., 2020; Kumar et al., 2024). The first fully annotated polyploid reference genome sequences of modern sugarcane cultivars R570 and ZZ1 have been recently published and are now publicly accessible (Healey et al., 2024; Bao et al., 2024). Given the role of the PP2C gene family in stress tolerance, understanding its evolutionary conservation, divergence, and functional significance in sugarcane hybrid cultivars is crucial for addressing knowledge gaps in its biological functions. This study provides a comprehensive bioinformatics analysis for the genome-wide identification and characterization of the PP2C gene family in sugarcane, including assessments of their physical and chemical properties, phylogenetic relationships, collinearity, gene structure, conserved motifs, chromosome mapping, and subcellular localization. The findings provide a foundational framework for further functional exploration of PP2C genes in polyploid sugarcane, offering valuable insights for future breeding efforts aimed at crop improvement.

## Materials and methods

### Genome-wide identification of PP2C genes in sugarcane

The whole genome, gene, and protein sequences of the polyploid sugarcane cultivar R570 were downloaded from the Sugarcane Genome Hub. To identify PP2C genes in sugarcane, the signature PP2C domains from rice, barley, sorghum, and maize were obtained from the EKPD database (Wang et al., 2014). Additionally, the amino acid sequences of all reported PP2C genes from *S. spontaneum* were considered for identification (Huang et al., 2023). For the identification of candidate PP2C family protein sequences in sugarcane, multiple sequence alignment of all reported PP2C proteins from rice, maize, barley, *S. spontaneum*, and sorghum was performed using T-Coffee (Notredame et al., 2000). Subsequently, an HMM search was conducted on the whole-genome protein sequence of polyploid sugarcane cultivar R570 using the PP2C domain. Significant hits with an E-value <0.01 were further verified for the presence of the PP2C domain using Pfam and Conserved Domain Database (CDD) (Bateman et al., 2004; Marchler-Bauer et al., 2015). Finally, to maintain a uniform identification within the group, nomenclature was assigned to all identified PP2C genes in sugarcane hybrid cultivar R570 based on their chromosomal positions.

### Phylogenetic study and ortholog analysis of PP2C genes in sugarcane

For evolutionary analysis, the protein sequences of identified PP2C genes in the sugarcane hybrid cultivar were aligned with PP2C protein sequences from maize, wheat, and *S. spontaneum* using T-Coffee with default parameters. A phylogenetic tree was constructed based on this alignment using the Maximum Likelihood (ML) method in MEGA11 with default parameters (Tamura et al., 2021). For comparison with a model organism, an additional phylogenetic analysis was performed using Arabidopsis PP2C genes in MEGA11 with 500 bootstrap replications. The generated tree was then annotated using iTOL (Letunic and Bork, 2024). To investigate the evolutionary relationship and genetic conservation of PP2C genes in sugarcane, a protein BLAST-based ortholog search was conducted using the PP2C protein sequences identified in the sugarcane cultivar R570. These sequences were mapped against its wild progenitors, *S. officinarum* and *S. spontaneum*.

### Identification of basic features, conserved motifs and domains, gene structure, and subcellular localization

The basic molecular features of PP2C proteins, including molecular weight, isoelectric point, and protein sequence length, were determined using the ProtParam tool (Gasteiger et al., 2003). Conserved motifs were identified through the MEME Suite (Bailey et al., 2009), with the number of motifs set to 10, while other parameters were kept at default. The exon-intron structure of the identified PP2C genes was analyzed using the Gene Structure Display Server (GSDS) (Guo et al., 2007). Subcellular localization predictions were performed using CELLO2GO (Yu et al., 2014). Conserved domains of PP2C proteins were predicted using the InterPro database (Hunter et al., 2009), and the results were visualized using TBtools (Chen et al., 2020) to provide a clear representation of domain structures.

### Chromosomal location, collinearity, gene duplication analysis and evolutionary selection pressure

The genomic features of all identified PP2C genes were extracted from the annotation file. Chromosomal mapping of these genes was performed using TBtools (Chen et al., 2020). Synteny analysis was conducted between the polyploid sugarcane cultivar and wild sugarcane (*S. spontaneum* AP85-441) using the Blastall program (Madden, 2013), followed by MCScanX (Wang et al., 2012). The synteny network was further visualized using Cytoscape (Kohl et al., 2011). A Circos plot was generated using TBtools (Chen et al., 2020) to illustrate tandem duplication among the identified PP2C genes in sugarcane. All collinear genes were analyzed for conserved domains, which were predicted using the InterPro database (Hunter et al., 2009) and visualized using TBtools (Chen et al., 2020). Evolutionary selection pressure among duplicated SoffiXPP2C gene pairs was assessed by calculating the non-synonymous (Ka) and synonymous (Ks) substitution rates using TBtools.

### Homology search with differentially expressed contigs and gene ontology analysis

CLC Genomics Workbench was utilized to analyze in-house generated RNA-Seq data from the sugarcane hybrid cultivar Co 86032 to identify differentially expressed genes under control and oxidative stress conditions. A *de novo* transcriptome assembly approach was employed for the analysis. A total of 37,356 contigs were identified as differentially expressed. To investigate potential homology with PP2C genes, a pairwise alignment was performed between the FASTA sequences of these differentially expressed genes and 500 PP2C genes identified in sugarcane in the current study. Out of these, 34 SoffiXPP2C genes were found to align with five differentially expressed contigs. Further functional annotation of these five differentially expressed contigs was performed using UniProt. Gene Ontology (GO) analysis was carried out for 34 SoffiXPP2C genes. Pairwise sequence alignment using BLASTp was conducted between these 34 genes and the protein sequences from rice and sorghum. The GO terms (GO IDs) associated with the rice and sorghum homologs identified through BLAST were retrieved from Oryzabase and SorghumBase. These GO terms were then putatively assigned to the corresponding SoffiXPP2C genes based on sequence homology.

### PCR amplification of SoffiXPP2C genes in genomic DNA and waterlogging stressed cDNA

To confirm the presence of four selected PP2C genes *SoffiXPP2C31*, *SoffiXPP2C75*, *SoffiXPP2C60*, and *SoffiXPP2C14* in sugarcane, PCR amplification was performed using genomic DNA as the template. Genomic DNA was extracted from leaf samples of five sugarcane cultivars (Co 86032, Co 11015, Co 91010, Co 0233, and Co 16001) using a standard DNA extraction method (CTAB). PCR amplification was performed in 20 μl reaction volumes containing PCR Master Mix, primers, and genomic DNA template. Amplified products were separated on 1% agarose gels stained with ethidium bromide and visualized under UV light.

Further to evaluate its expression in stress condition all this SoffiXPP2C genes (*SoffiXPP2C31*, *75*, *60*, and *14*) were amplified in waterlogged stressed cDNA. The waterlogging condition in sugarcane genotypes was conducted in the green house at Sugarcane Research Station, Cuddalore. Three Clones/varieties of sugarcane like CoC 13339, C 16338 and C 2014-516 compared with tolerant (Co 62175) and susceptible (Co 86032). All plants were grown under well-watered but non-flooded conditions during the first one and a half months for proper establishment. Forty-five days old seedlings were subjected to waterlogging for twenty days continuously by maintaining the water level at 10cm above soil level. Simultaneously control pots were well watered (without waterlogging) and maintained with normal cultural practices.

The appropriate root and stem samples of Co 62175 (1), Co 86032 (4), CoC 13339 (5), C 16338 (8) and C 2014-516 (11) were collected from the field and stored in RNA later storage solution. The required materials for the RNA isolation were treated with 0.1% DEPC for overnight at 37°C, subsequently double autoclaved at 121°C for 20 min. The collected samples were ground to fine powder using liquid nitrogen. Total RNA isolation was carried out using RNeasy Plant mini kit. Integrity and quality of the RNA samples were checked by 1% (w/v). The quality and quantity of RNA were assessed using the Nanodrop spectrophotometer.

### cDNA conversion and RT-qPCR

The PrimeScriptTM 1st strand cDNA synthesis kit (Takara Bio) was used for cDNA conversion. cDNA was quantified using nanodrop 2000 and diluted 100 ng/μL. The 25S rRNA gene was used as a reference for the stress responsive gene expression analysis. The normalization of cDNA samples performed using 25S rRNA primer. Quantitative PCR was performed for all the selected genes and also for the reference gene using TB GREEN^®^ premix Ex Taq™ II mix (TAKARA). Each reaction volume of 20 μL consists of 100 ng cDNA, TB GREEN master mix, 10 μM each of forward and reverse primers and nuclease-free water to make up the volume. The PCR cycle included a pre-incubation at 95°C for 1 minute followed by 40 cycles of amplification at 95°C for 5 seconds and required Tm for 30 seconds acquiring on cycle A Green channel and for Melt A Green ramp selected between 50°C -60°C. The Gain optimisation was selected at 1 to 3 range for Green channel.

The RT-qPCR experiments were performed using the Rotor-GeneQ Qiagen Real-Time PCR system (Qiagen, Hilden, Germany) and SYBR Green PCR Kit (TAKARA). The relative expression of gene in relation to stem, root & reference gene (25S RNA) are calculated on the basis of ‘delta delta Ct’ (ΔΔCt) values (Livak and Schmittgen, 2001). The most commonly used method for relative quantification is the 2(–ΔΔCt) method. Exported the raw Ct values (CT1 and CT2) from the real-time PCR analysis into Microsoft Excel sheet. Assigned sample number, gene name to each data set. Calculated the mean Ct value for the internal control gene and sample gene. The mean fold change (ΔΔCt), 2(–ΔΔCt) and standard deviation were calculated and interpreted in a graph with standard error values.

The equation as follows,


ΔCT=Mean CT of Sample gene–Mean CT of Reference gene

ΔΔCT=ΔCT of each sample−Average ΔCT of all samples

Fold expression=2−ΔΔCT

## Results

### Genome wide identification and phylogenetic study and ortholog analysis of PP2C genes in sugarcane

A total of 500 PP2C (Protein Phosphatase 2C) genes were identified in the sugarcane genome, with their respective gene IDs listed in **Supplementary Table S1**. To identify these genes, multiple sequence alignment was performed with T-Coffee using the PP2C signature domain from *S. spontaneum*, rice, barley, sorghum, and maize, followed by an HMM search using the whole-genome protein sequence of the sugarcane under study. Sequences lacking complete conserved domains associated with the PP2C superfamily were excluded from further analysis. Ultimately, 500 genes of sugarcane hybrid cultivar under study containing the PP2C signature domain were selected for downstream analysis. In order to keep uniform identification, all these genes were renamed as SoffiXPP2C where SoffiX represents “*Saccharum officinarum* and *Saccharum spontaneum hybrid”* and PP2C represents “Protein Phosphatase 2C”, followed by a unique number assigned based on their order of position on the chromosome.

Phylogenetic analysis of the PP2C genes was performed using T-Coffee for multiple sequence alignment and MEGA 11 for tree construction, with PP2C genes from *S. spontaneum*, wheat, and maize as reference. Based on this analysis, all 500 genes were classified into 13 subfamilies (A–M) (**Supplementary Table S2**). The distribution of the gene within its subfamily was uniform in the phylogenetic analysis involving sugarcane and wheat (**Supplementary Figure S3**), as well as sugarcane and *S. spontaneum* (**Supplementary Figure S4**). Gene subfamily assignments were made based on the combined results of these phylogenetic analyses.

**Figure 1:**
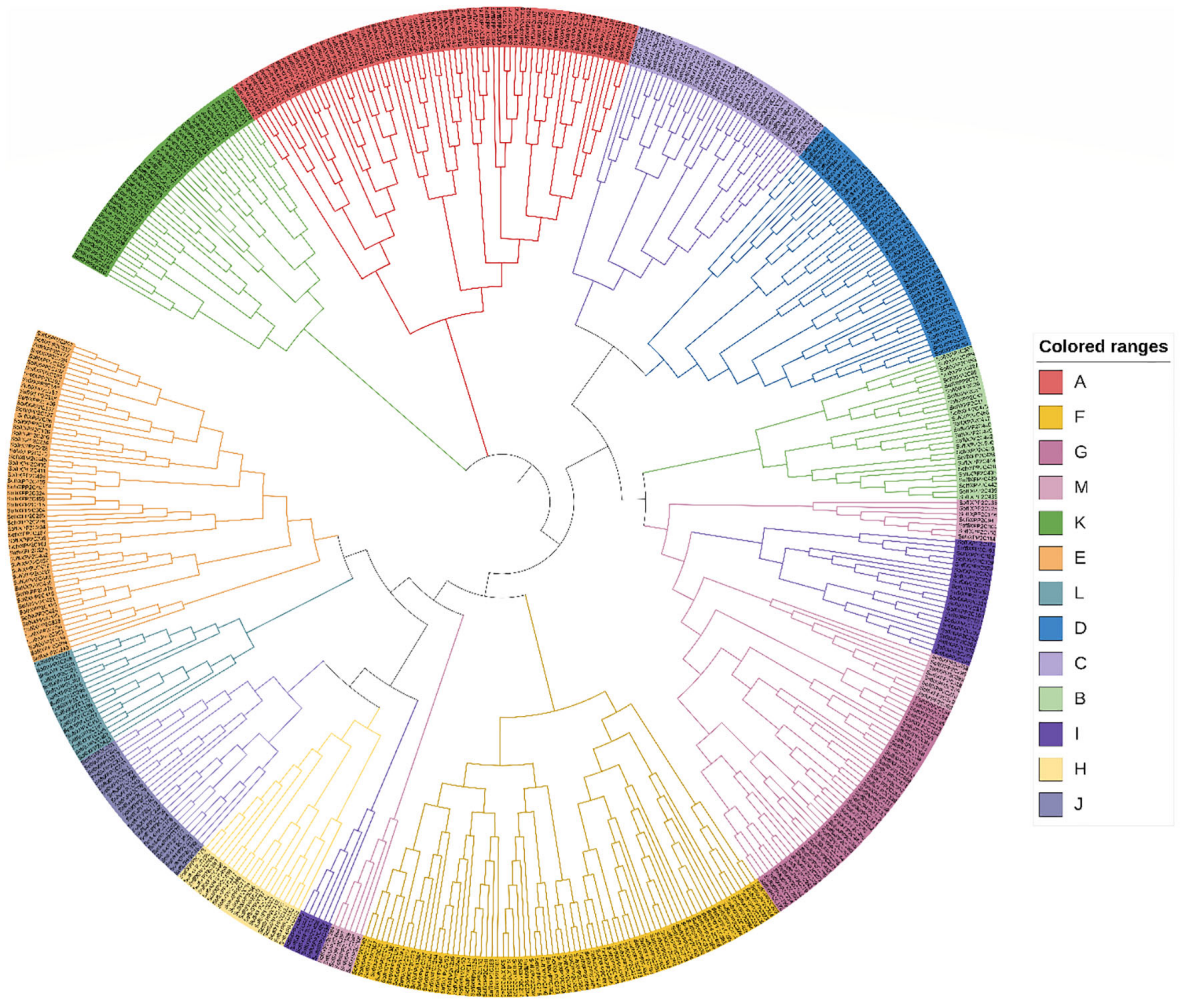
Phylogenetic relationship among the 500 SoffiXPP2C genes. The phylogenetic tree was constructed using MEGA 11 with the ML method and default parameters. The SoffiXPP2C genes were classified into 13 subgroups **(A–M)**, each represented by a distinct color. The color denoting each subfamily is provided on the right side of the tree. SoffiXPP2C stands for “*Saccharum officinarum* and *Saccharum spontaneum hybrid”* and PP2C represents “Protein Phosphatase 2C.

Finally, a phylogenetic tree has been made considering only SoffiXPP2C genes (**Figure 1**). The distribution of gene in phylogenetic tree has shown that all the genes were clustered together of assigned subfamily in previous analysis, except few. Among 13 subfamily largest subfamily was F with 74 genes, and smallest one was L with 18 genes. Subfamily A was second largest subfamily with 69 genes followed by E, D, G, K, C, I, B, J, H, and M which contains 57, 44, 39, 35, 27, 26, 25 and 21 genes respectively. Subfamily D and G contains equal number of genes i.e., 44 and subfamily H and M also contains equal number of genes i.e., 21. Additionally, phylogenetic analysis of 500 sugarcane PP2C genes was carried out using the reported PP2C genes of *Arabidopsis thaliana* (**Supplementary Figure S5**). All sugarcane genes followed the previously established classification pattern of Arabidopsis and were clustered into 13 subfamilies, as reported by Xue et al. (2008). The distribution of genes among the subfamilies was largely consistent with prior classifications based on analysis with *S.* *spontaneum* PP2C genes. Only a few genes were found to be clustered differently in the analysis with Arabidopsis compared to that with *S. spontaneum*.

In the ortholog analysis, all PP2C genes identified in Sugarcane R570 showed significant identity to genes in both wild species, *S. officinarum* and *S. spontaneum* (**Supplementary Figure S6**). Out of the 500 identified PP2C genes in R570, 215 genes (43%) showed 100% identity with *S. officinarum*, whereas only 14 genes showed 100% identity with *S. spontaneum*. Notably, 9 of these genes were common to both wild species. A total of 134 genes exhibited 99% identity with *S. officinarum*, while 110 genes showed 99% identity with *S. spontaneum*, with 43 genes being shared. Additionally, 75 genes displayed 98% identity with *S. officinarum*, while 114 genes showed the same identity level with *S. spontaneum*, and 16 of these were common to both. In the identity range of 50–90%, 20 genes matched with *S. officinarum*, whereas 112 genes matched with *S. spontaneum*, including 43 overlapping genes. No genes have detected identity less than 52% with S. officinarum, whereas up to 30% identity was observed with S. *spontaneum*.

### Identification of basic features, conserved motifs and domains, gene structure, and subcellular localization

For the identified PP2C genes in sugarcane, key physical and biochemical properties were predicted, including gene and protein length (amino acids), molecular weight, isoelectric point (pI), and subcellular localization (**Supplementary Table S1**). Gene length was determined from genome annotation files (GFF). The longest gene identified was *SoffiXPP2C267* spanning 36,077 bp, while the shortest was *SoffiXPP2C249*, measuring 305 bp. The longest protein SoffiXPP2C436, consisting of 1,119 amino acids, whereas the shortest proteins, SoffiXPP2C242 and SoffiXPP2C249, each contained 101 amino acids. The highest molecular weight was observed in SoffiXPP2C436 at 127.29 kDa, while the lowest was in SoffiXPP2C249, measuring 10.96 kDa. The highest pI was recorded for SoffiXPP2C423 at 11.14, whereas the lowest was found in SoffiXPP2C213, at 0.13. The CDD algorithm confirmed the presence of the PP2C domain in all SoffiXPP2C members. Notably, the localization of this domain was highly conserved across various PP2C subfamilies.

The analysis of gene structure, in terms of exon numbers and exon-intron distribution, revealed a high degree of conservation within each SoffiXPP2C subfamily (**Supplementary Figure S1**). The distribution of exons varied among the subfamilies, with subfamily A and C containing 1, 3, and 4 exons, while subfamily B had 1, 3, 4, and 5 exons. Similarly, subfamily D showed a distribution of 1, 2, 4, and 7 exons. Subfamily E contained 1 to 6 exons, and subfamily F exhibited a broader range, with 2 to 10 exons. The exon distribution in subfamily G included 3, 4, 10, and 12 exons, while subfamily H had 7 and 8 exons. Subfamily I displayed significant variation, with 10, 11, and 20 exons, whereas subfamily J had 3, 8, and 9 exons. In subfamily K, the exon count ranged from 1 to 12, and subfamily L had 3 and 14 exons. Subfamily M exhibited exon numbers ranging from 6 to 13. The highest exon count (20 exons) was observed in *SoffiXPP2C436*, *SoffiXPP2C491*, and *SoffiXPP2C441*, while the minimum exon count (1 exon) was found in 37 genes. A total of ten motifs were identified in the SoffiXPP2C genes using the MEME tool (**Supplementary Figure S2**). Among them, Motifs 1, 3, 4, and 6 were present in all 500 SoffiXPP2C genes. Certain subfamilies exhibited identical motif compositions, with subfamilies A, E, F, G, I, and M sharing a completely similar motif pattern. Similarly, subfamilies H and J, as well as subfamilies C and L, displayed identical motif distributions. Notably, Motif 9 was exclusively present in subfamilies H and J, whereas Motif 7 was uniquely found in subfamilies B, H, and J.

The predicted subcellular localization of PP2C proteins indicated their predominant presence in the chloroplast (213 proteins), followed by the cytoplasm (156 proteins) and the nucleus (81 proteins). Analysis of conserved domains showed that sugarcane PP2C domain-containing proteins exhibit diverse structural features, including PP2C catalytic domains, PPM-type phosphatase domains, and PP2C signature motifs, which are present in almost all proteins. Additionally, several proteins also contain kinase domains, signal peptides, transmembrane regions, membrane-associated regions, extracellular domains, hydrophobic regions, disordered regions, and potential phosphorylation sites, highlighting their structural and functional complexity.

### Chromosomal localization, synteny, gene duplication analysis and evolutionary selection pressure

**Figure 2:**
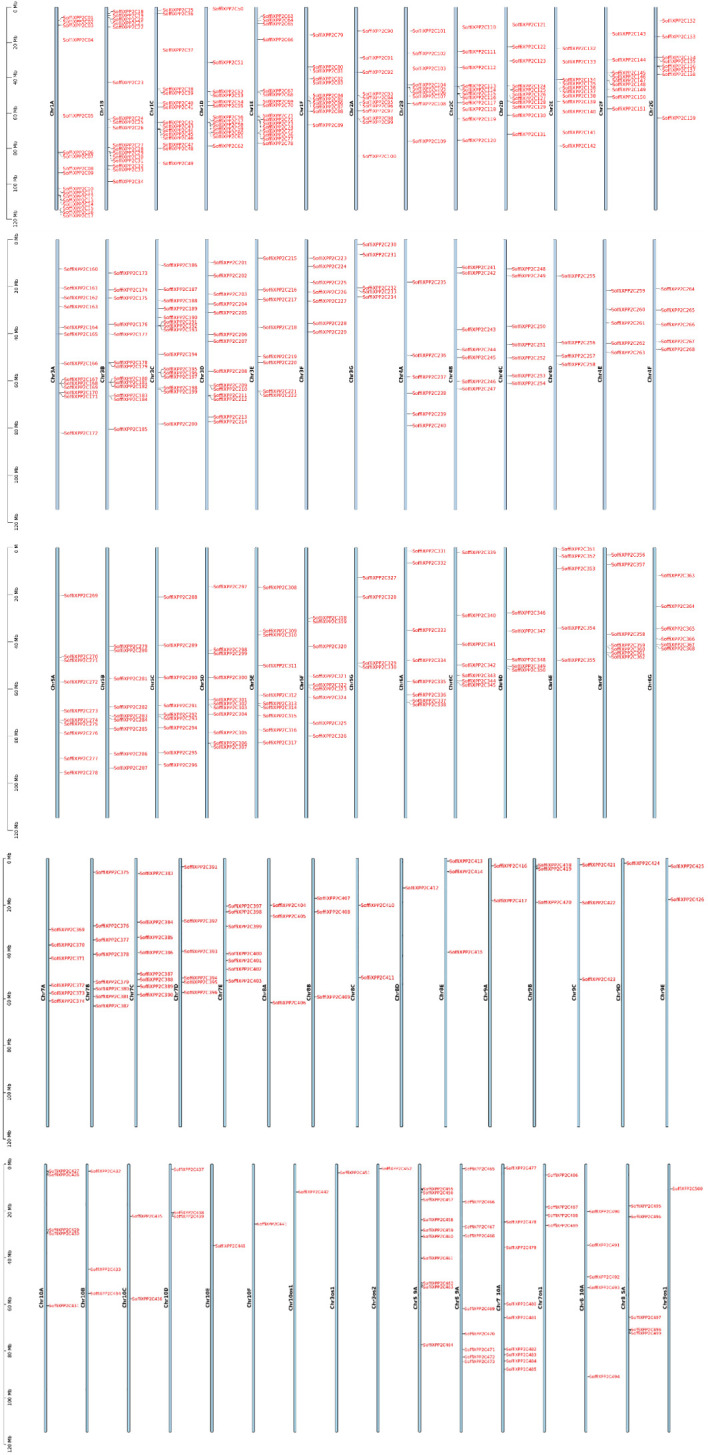
Chromosomal distribution of SoffiXPP2C genes in sugarcane. Bar represent chromosome with chromosome names displayed vertically in the center. Chromosomes are arranged in order, and each SoffiXPP2C gene is mapped to its respective chromosomal position. SoffiXPP2C stands for “*Saccharum officinarum* and *Saccharum spontaneum hybrid”* and PP2C represents “Protein Phosphatase 2C.

All 500 PP2C genes were identified and distributed across all 10 chromosomes and their sub genomes (**Figure 2**; **Supplementary Table S1**). The highest number of genes (89) was found on chromosome 1 and its subgenome, followed by chromosome 3 and its subgenome (76 genes) and chromosome 2 and its subgenome (70 genes). Chromosome 5, 6, 7, 4, 8, 9 and 10 along with their subgenome contained 62, 38, 36, 34, 12, 11, 15 genes respectively. Additionally, genes were identified in various interspecific recombinant chromosome regions, including Chr5_9A (10 genes), Chr6_9A (9 genes), Chr7_10A (9 genes), Chr7os1 (4 genes), Chr8_10A (5 genes), and Chr8_5A (5 genes), while the lowest number of genes (1) was found on the chromosomes 10os1, 3os1, 3os2, and 9os1. In addition, 13 genes were identified in various scaffolds.

**Figure 3:**
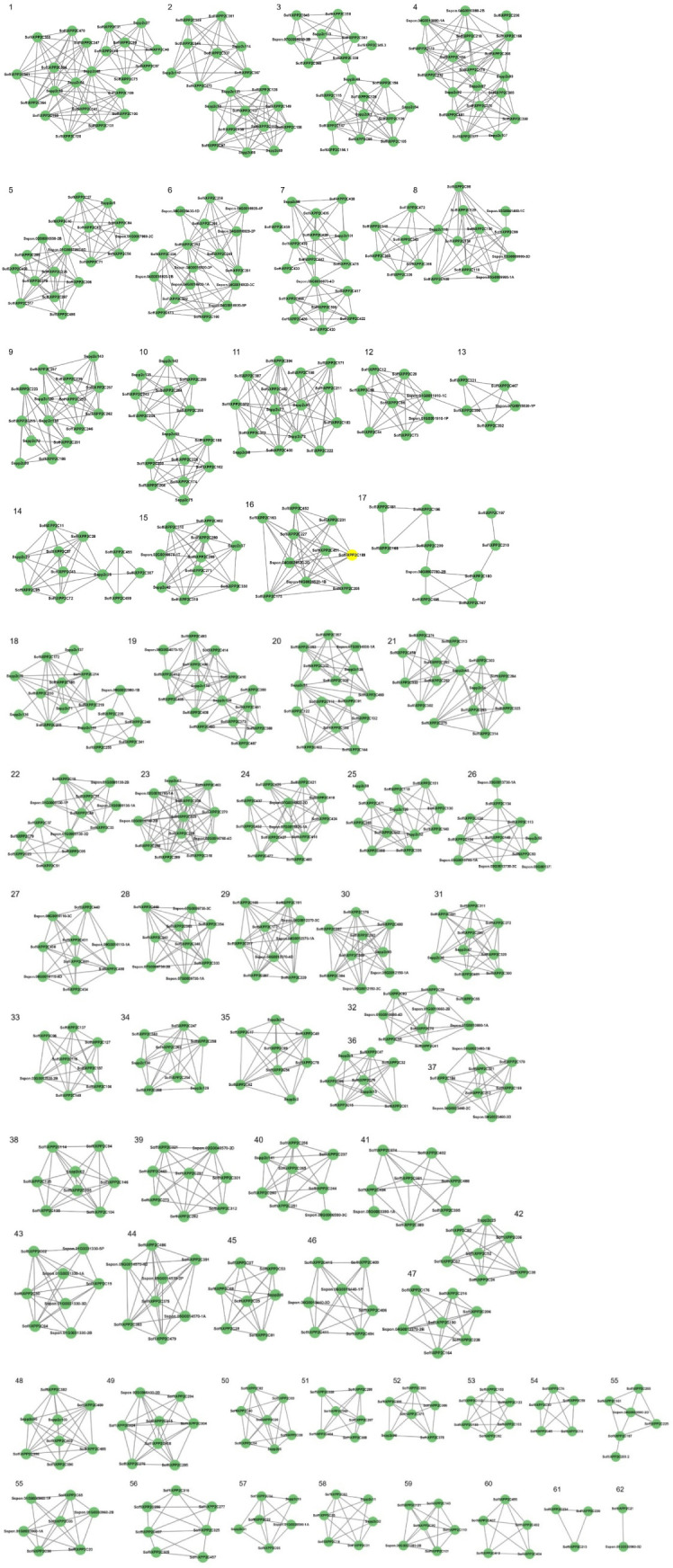
Synteny network for SoffiXPP2C genes in sugarcane and genes of *S. spontaneum*. Here nodes represent gene and edge represent syntenic association between them. SoffiXPP2C, *Saccharum officinarum* and *Saccharum spontaneum hybrid”* and PP2C represents “Protein Phosphatase 2C; Sspp2c, *Saccharum spontaneum* PP2C; Sspon, *Saccharum spontaneum*.

The characterization of the PP2C gene family in *S. spontaneum* identified PP2C genes exhibiting cold-stress-induced expression (Huang et al., 2023). Additionally, various studies have reported that wild sugarcane (*S. spontaneum)* is a drought-tolerant grass (Mirshad and Puthur, 2017). To understand the collinearity and gene conservation patterns of PP2C genes between the sugarcane hybrid cultivar genome under study and *S. spontaneum*, a synteny analysis was performed (**Figure 3**; **Supplementary Table S3**). A total of 633 genes were found to be involved in synteny relationships, forming 1,988 pairs of associations and clustering into 62 synteny networks. Among these synteny pairs, 477 out of 500 PP2C genes were found to be associated with other genes from the sugarcane hybrid cultivar under study and *S. spontaneum*. A total of 156 genes from *S. spontaneum* were involved in synteny associations, of which 76 genes were identified as PP2C genes. All *S. spontaneum* genes involved in synteny relationships were located on five chromosomes and their subgenomes (1, 2, 3, 4, and 5). The gene IDs of PP2C genes in *S. spontaneum*, along with their locus IDs involved in synteny relationships, are listed in **Supplementary Table S4**. Furthermore, in the sugarcane hybrid cultivar, all PP2C genes with chromosome annotations were included in the synteny analysis and found to be involved in synteny relationships.

**Figure 4:**
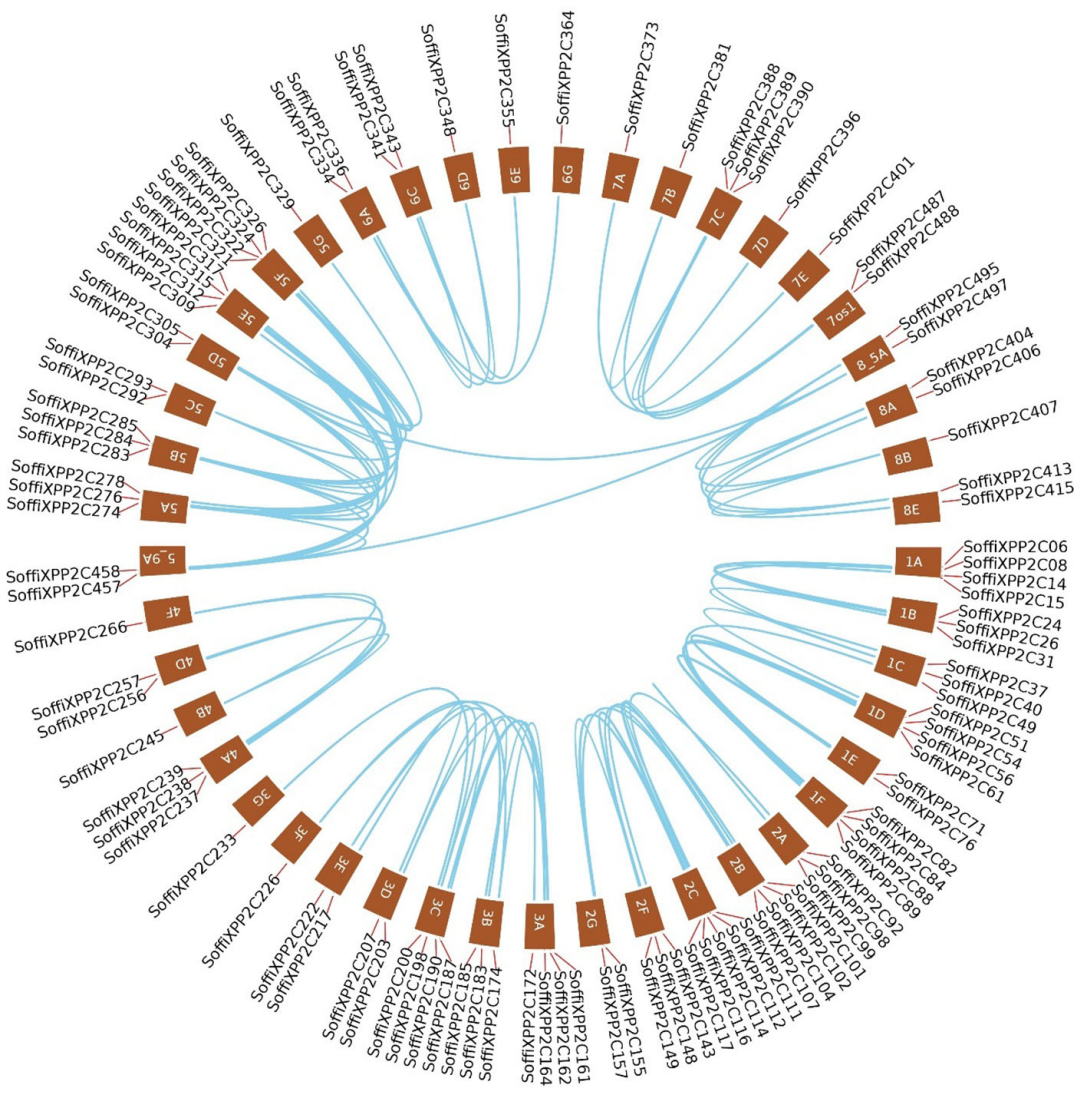
Gene duplication analysis of the PP2C gene family in sugarcane. The labelled bars represent sugarcane chromosomes with their associated genes. Blue lines indicate paralogous relationships between respective SoffiXPP2C genes, highlighting gene duplication events. SoffiXPP2C stands for “*Saccharum officinarum* and *Saccharum spontaneum hybrid”* and PP2C represents “Protein Phosphatase 2C.

Gene duplication events were analyzed to identify tandem duplication patterns in the PP2C gene family of sugarcane. Out of 500 genes, 107 were identified as duplicated, indicating the presence of paralogous genes (**Figure 4**). A total of 105 duplicated genes were mapped across 45 chromosomes, including 8 chromosomes and their subgenomes, along with 5_9A, 8_5A, and 7os1. Additionally, two duplicated genes were found in scaffolds. Among the PP2C subfamilies, subfamily F exhibited the highest number of duplication events, with 23 genes involved, followed by subfamily E (18 genes), A (11 genes), D (11 genes), G (10 genes), H (8 genes), K (8 genes), I (5 genes), J (5 genes), M (5 genes), and L (3 genes).

Pairwise synonymous (Ks) and non-synonymous (Ka) substitution rate analysis was performed among duplicated SoffiXPP2C gene pairs, and the results indicated that most gene pairs showed Ka value 0, while Ks values were 0 or less than 1 (**Supplementary Table S8**). Only two pairs had non-zero substitution ratios such as “*SoffiXPP2C82* and *SoffiXPP2C54*” with a Ka/Ks value of 0.6759, and “*SoffiXPP2C89* and *SoffiXPP2C49*” with a value of 0.12595. No gene pairs exhibited a Ka/Ks ratio.

### Homology search with differentially expressed contigs and gene ontology analysis

A total of 34 out of 500 PP2C genes from the sugarcane hybrid cultivar were found to align with five differentially expressed contigs under oxidative stress conditions. **Supplementary Table S5** presents these 34 SoffiXPP2C genes along with their alignment details with the five differentially expressed contigs. **Supplementary Table S6** presents the differential expression data for five contigs that showed homology with 34 SoffiXPP2C genes in sugarcane hybrid cultivar. It details expression metrics such as Max Group Mean, Log_2_ Fold Change, Fold Change, P-value, FDR P-value, and Bonferroni correction. Among these, Contigs joined_130 (SoffiXPP2C95, 105, 115, 126, 136, 142, 147, 156, and 351) exhibited the highest expression level (Max Group Mean = 17.11) with a Log_2_ Fold Change of 0.6 and a statistically significant P-value of 0.00434. In contrast, Contigs joined_733 (SoffiXPP2C142) displayed a negative Log_2_ Fold Change (-4.02) and a large negative Fold Change (-16.21), indicating strong downregulation. Contigs joined_346 (SoffiXPP2C10, 27, 42, 56, 71, 84, 278, 287, 296, 306, 317, 326, 448, 456 and 498) showed moderate upregulation with a Log_2_ Fold Change of 0.71. On the other hand, Contigs joined_36 (SoffiXPP2C161, 187, 2023, 225) and Contigs joined_215 (SoffiXPP2C332, 339, 353, 357, 468, and 474) had a Max Group Mean of 0 and contained NaN values for differential expression metrics, indicating no detectable significant expression changes. The presence of NaN values suggests missing or unmeasurable expression levels, which may require further validation. Further annotation from UniProt revealed that four out of five differentially expressed contigs are associated with functions related to protein-serine/threonine phosphatases and the phosphatase 2C family. Additionally, they were identified as PPM-type phosphatase domain-containing proteins, RRM domain-containing proteins, splicing factor U2AF large subunit, and poly(A)-specific ribonuclease, while one contig was identified as glycerol kinase (**Supplementary Table S6**).

**Figure 5:**
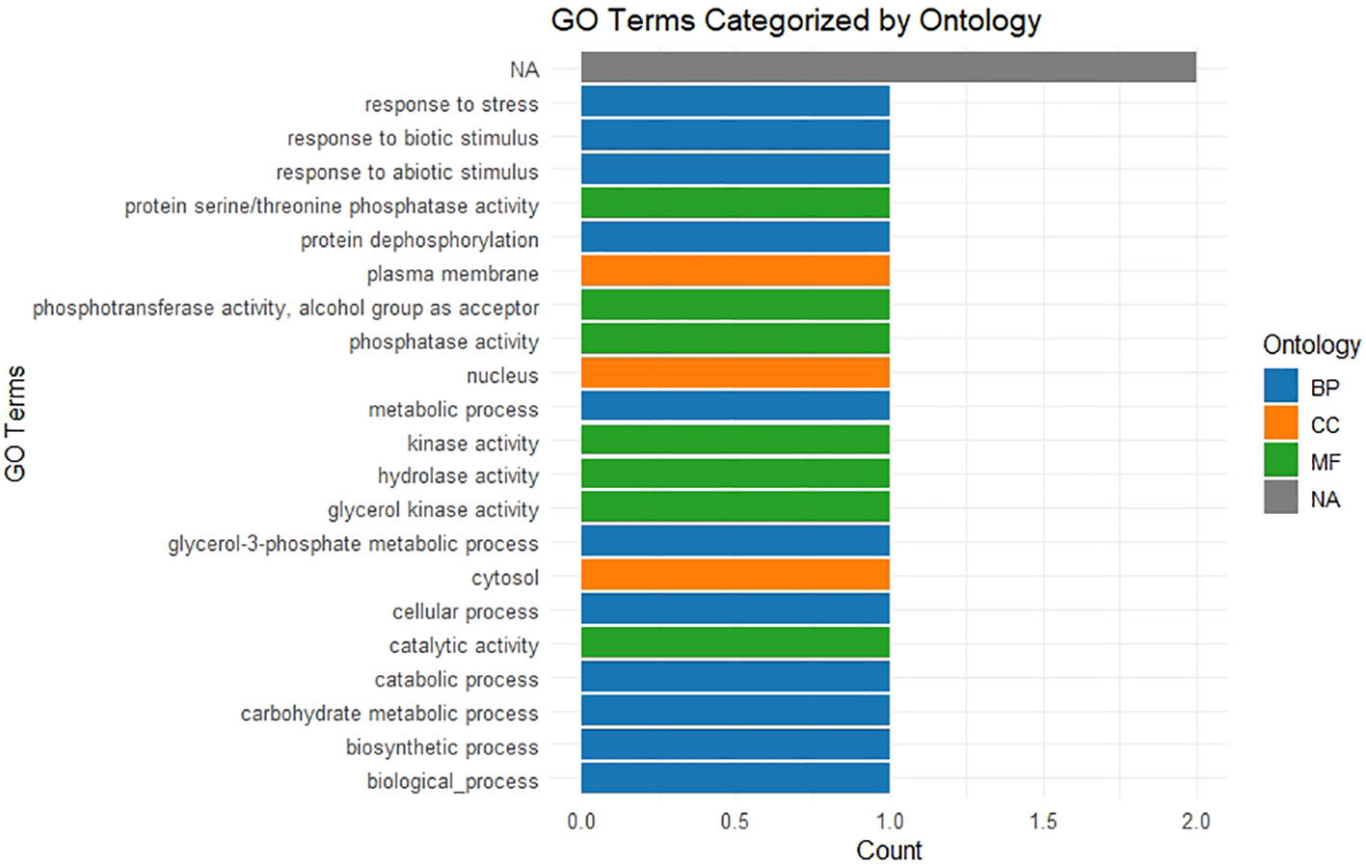
Gene ontology (GO) analysis of SoffiXPP2C genes exhibiting homology with differentially expressed genes. BP, Biological Process (blue); MF, Molecular Function (green); CC, Cellular Component (orange); NA, Not Assigned or Uncategorized (gray).

Gene Ontology (GO) analysis was conducted for 34 SoffiXPP2C genes based on their sequence homology with rice and sorghum (**Supplementary Table S7**). Six homologous genes from each of rice and sorghum were identified corresponding to the SoffiXPP2C gene set. The GO terms associated with these homologs were retrieved and used to infer the putative functions of the SoffiXPP2C genes. GO term assignments for mapping of functional annotations to the GO terms were performed using the Go.db package in R (**Figure 5**). A large number of Gene Ontology (GO) terms fall under the biological Process (BP) category, indicating functional roles in response to stress, biotic and abiotic stimuli, metabolic processes (including carbohydrate metabolism, glycerol-3-phosphate metabolism, and biosynthetic pathways), as well as catabolic and general cellular processes. In the Molecular Function (MF) category, the GO terms suggest involvement in phosphatase activity (protein serine/threonine phosphatase, hydrolase, and kinase activities) along with catalytic and transferase functions. For the Cellular Component (CC) category, the associated genes are primarily localized to the nucleus, cytosol, and plasma membrane.

### PCR amplification of PP2C genes in genomic DNA and waterlogging stressed cDNA and RT-qPCR

**Figure 6:**
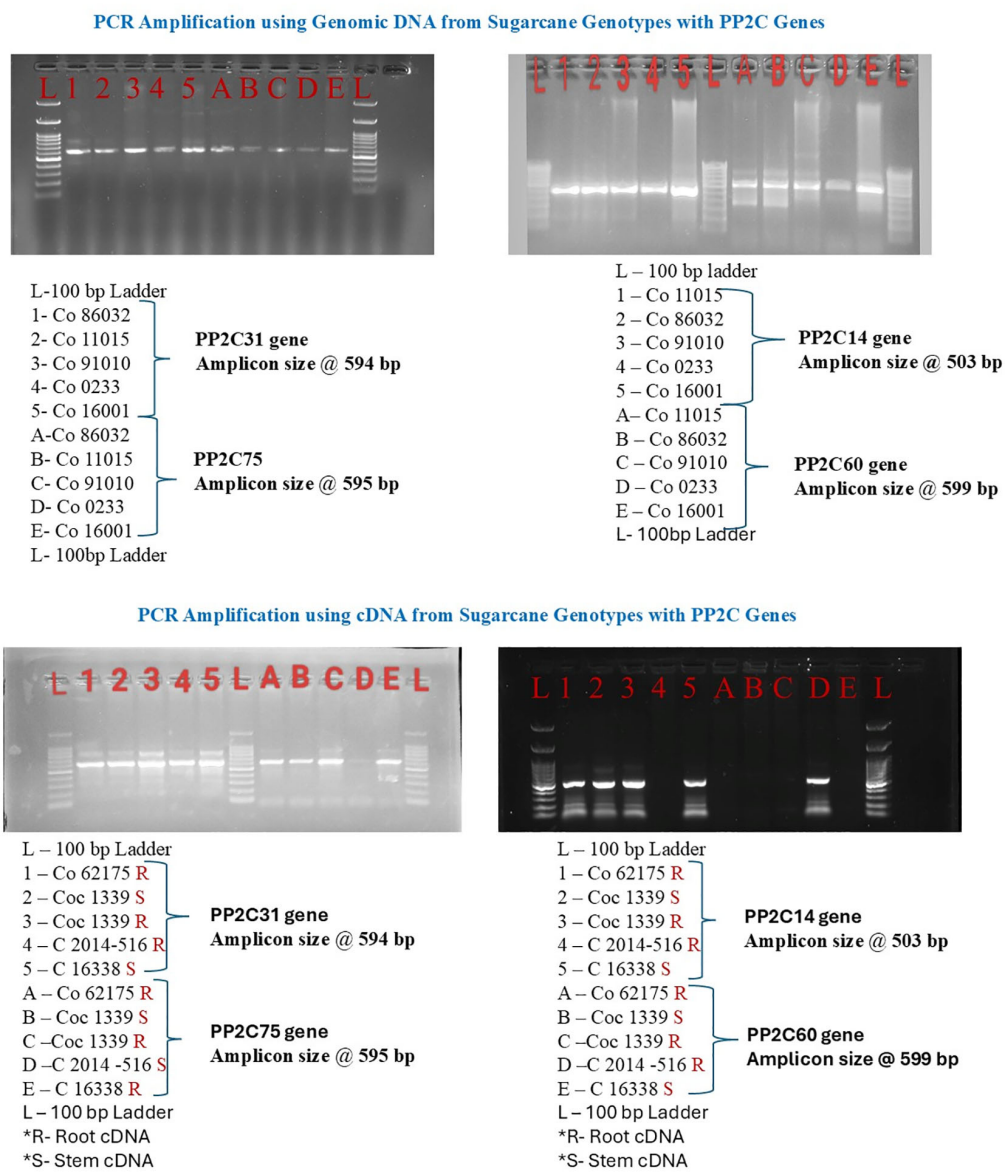
PCR amplification of four PP2C genes (*SoffiXPP2C31*, *SoffiXPP2C75*, *SoffiXPP2C60*, and *SoffiXPP2C14*) in genomic DNA and cDNA in sugarcane cultivar.

PCR amplification of four sugarcane PP2C genes *SoffiXPP2C31*, *SoffiXPP2C75*, *SoffiXPP2C60*, and *SoffiXPP2C14* was performed to confirm the presence of the target genes in genomic DNA and to assess their expression under waterlogging stress conditions using cDNA synthesized from treated samples (**Figure 6**). For genomic DNA amplification, leaf samples from five sugarcane cultivars Co 86032, Co 11015, Co 91010, Co 0233, and Co 16001 were used. All four genes showed successful amplification across all cultivars producing bands of different sizes: *SoffiXPP2C31* (594 bp), *SoffiXPP2C75* (595 bp), *SoffiXPP2C14* (503 bp), and *SoffiXPP2C60* (599 bp), confirming their presence in the genome. For cDNA amplification, the stem and root samples from five sugarcane cultivars Co 62175, Coc 1339, Coc 1339, C 2014 516, and C 16338 were analysed. *SoffiXPP2C31* showed amplification in both stem and root samples of all cultivars producing bands of 594 bp. *SoffiXPP2C75* was amplified in all samples except the stem cDNA of cultivar C 2014 516 producing bands of 595 bp. *SoffiXPP2C14* showed amplification in all samples except the root cDNA of cultivar C 2014 516 producing bands of 503 bp. *SoffiXPP2C60* amplification was observed only in the root cDNA of cultivar C 2014 516 producing bands of 599 bp (**Figure 6**).

Gene expression profiling of roots and stem samples of sugarcane was conducted through qRT-PCR. The samples were taken from the waterlogging stress experiments. The relative expression levels of *SoffiXPP2C75, SoffiXPP2C60, SoffiXPP2C31* and *SoffiXPP2C14* genes with the endogenous control 25s were examined using relative quantification or comparative CT method. Fold expression [2^ (-ΔΔC T)] of the samples related to respective genes are interpreted (**Table 1**).

**Table 1:** Two-Fold expression value of SoffiXPP2C genes.

SAMPLE	PP2C75	PP2C60	PP2C31	PP2C14
1R	2.16059	3.53956	2.22556	0.72591
1S	2.55165	0.4693	2.10551	8.80216
4R	0.11961	0.58179	0.34608	0.58555
4S	0.12556	0.16824	0.29713	0.29379
5R	1.9405	1.77593	1.45818	0.23863
8R	0.13835	0.21148	0.35091	1.36401
11S	44.9868	16.3767	9.0265	2.79502

Genotype codes – Co 62175 (1), Co 86032 (4), CoC 13339 (5), C 16338 (8), and C 2014-516 (11). Tissue types – R, Root; S, Stem.

From the fold expression values of each sample both up regulated (greater than 1) and down (lesser than 1) regulated performance of gene has been observed.

**Figure 7:**
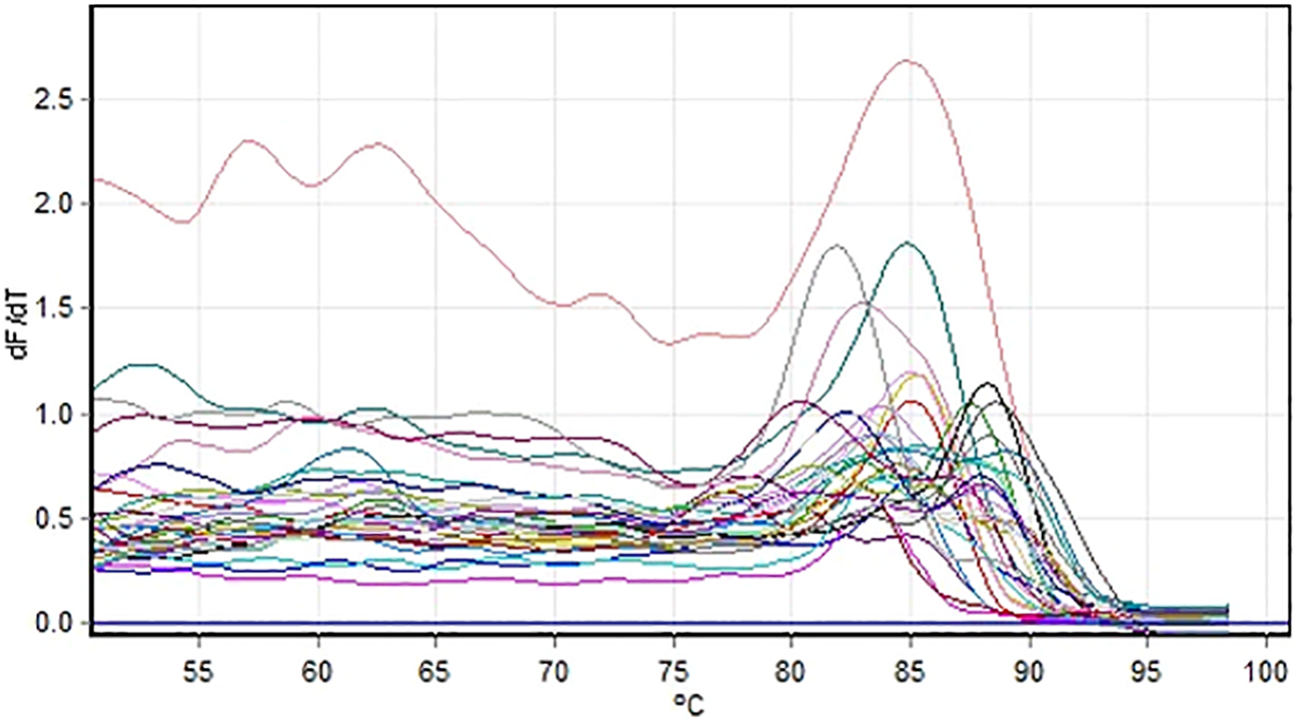
Melt curve of SoffiXPP2C genes studied for expression analysis using qRTPCR.

**Figure 8:**
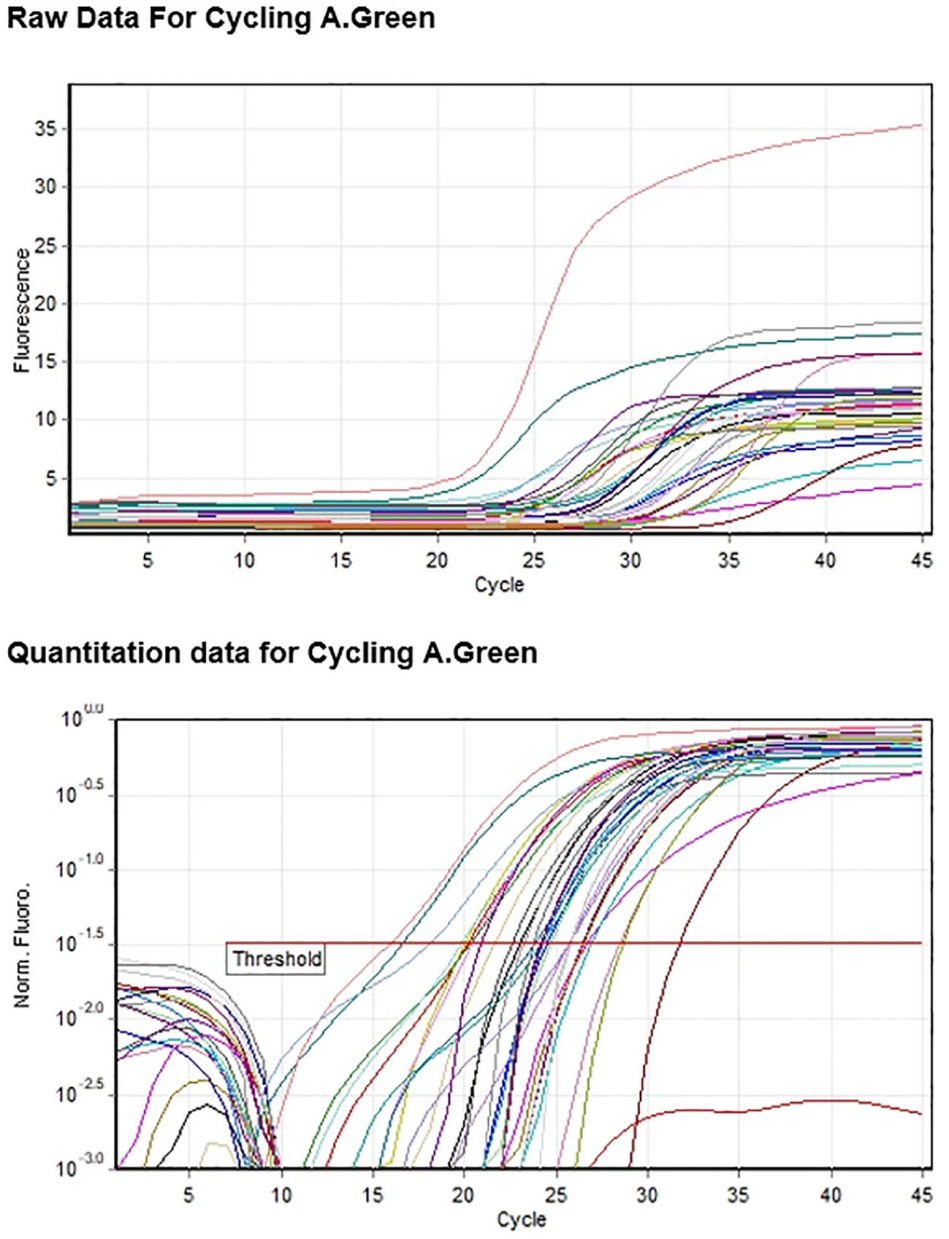
Amplification curve of SoffiXPP2C Genes studied for expression analysis using qRTPCR.

The root sample of Co 62175 (1R) showing up regulation for all the genes except *SoffiXPP2C14*. The stem sample of Co 62175 (1S) showing up regulation for all the genes except *SoffiXPP2C60*. The root and stem sample of this genotype showing relatively higher expression in *SoffiXPP2C60* and *SoffiXPP2C14* respectively. The root and stem sample of Co 86032 (4R & 4S) showing down regulation for all the four genes. The root sample of CoC 13339 (5R) up regulated in all genes except *SoffiXPP2C14*. The root sample of C 16338 showing up regulation in *SoffiXPP2C14* and all other genes are down regulated. The stem sample of C 2014-516 (11s) showing up regulation for all the four genes with higher expression in *SoffiXPP2C75* gene. The gene expression indicates that the *SoffiXPP2C* genes differentially expressed in different cultivars in root and stem samples (**Figures 7**, **8**).

## Discussion

PP2C phosphatases play a crucial role in abscisic acid (ABA) signaling, particularly in response to abiotic stress such as drought and salinity. These phosphatases act as negative regulators of SnRK2 protein kinases, which are essential for ABA-mediated stress responses. The interaction between PP2Cs and SnRK2s is pivotal for activation of downstream signaling pathways that enhance plant resilience to environmental stressors (Chuong et al., 2020; Sahoo et al., 2020; Chuong et al., 2021). PP2C phosphatases family is an ancient and highly conserved enzyme family, found across various life forms, including archaea, bacteria, fungi, plants, and animals (Fuchs et al., 2013). In Arabidopsis, 76 PP2C proteins have been identified, making it the largest protein phosphatase family in plants and underscoring their significance in stress management (Schweighofer et al., 2004; Xue et al., 2008).

In the present study, a whole-genome characterization of PP2C phosphatases was conducted in the modern polyploid sugarcane cultivar R570 which derived from inter-specific hybrids between *S. officinarum* and *S. spontaneum*. A total of 500 PP2C genes were identified and classified into 13 subfamilies (**Supplementary Tables S1**, **S2**). This high gene count is attributed to sugarcane’s polyploid nature but remains comparable to previous findings in other species, such as *Brassica* (131), soybean (134), *S. spontaneum* (145), Arabidopsis (80), rice (78), wheat (257), and maize (103) (Khan et al., 2019; Fan et al., 2020; Huang et al., 2023; Xue et al., 2008; Wu et al., 2023; Yu et al., 2019). Recently, a comprehensive analysis of the barley pan-genome revealed 1,635 PP2C genes across 20 accessions, including both wild and cultivated varieties, indicating a significant expansion of this gene family in barley (Wu et al., 2022). The expansion of the PP2C gene family in sugarcane, likely due to its complex polyploidy nature, highlights its evolutionary significance and genomic complexity (Leitch and Leitch, 2008; Soltis and Soltis, 2021). The number of PP2C genes varies significantly across plant groups, reflecting evolutionary adaptations. For instance, green algae such as *Chlamydomonas reinhardtii* possess only 10 PP2C proteins, while moss (*Physcomitrella patens*) has 50, and the flowering plant *Amborella trichopoda* contains 48. Interestingly, despite comparable genome sizes, Arabidopsis and rice contain 80 and 90 PP2C genes, respectively (Rensing et al., 2008; Merchant et al., 2007; Fuchs et al., 2013). In the current study, all 500 identified PP2C genes were classified into 13 subfamilies (A–M) (**Figure 1**; **Supplementary Table S2**). Subfamily F was the largest in sugarcane hybrid, with 74 members, mirroring findings in *S. spontaneum*, where subfamily F also had the highest member count (37 genes) (Huang et al., 2023). Subfamilies A and B members are absent in prokaryotes and non-plant eukaryotes. Interestingly, Clade A PP2Cs first appeared in unicellular green algae (*C. reinhardtii*), while Clade B PP2Cs are found in *Selaginella moellendorffii* and higher plants but are absent in *Chlamydomonas* and *Physcomitrella patens*. This variation suggests that as plants evolved from simpler to more complex structures, the PP2C gene family underwent significant expansion and diversification. The increase in PP2C gene count correlates with the evolution of multicellularity in plants, enabling them to better adapt to environmental changes (Xue et al., 2008; Fuchs et al., 2013; Huang et al., 2023).

Modern sugarcane is a polyploid, aneuploid, and interspecific hybrid with an estimated genome size exceeding 10 Gb. The majority of its genome is derived from *S. officinarum* (~80%) and *S. spontaneum* (10–15%), along with some recombinant chromosomes (5–10%) (Sharma et al., 2018; Dinesh Babu et al., 2022). To analyze the genomic contribution to the identified PP2C genes, an ortholog analysis was performed using BLAST against the genomes of *S. officinarum* and *S. spontaneum*. The results reveal a higher degree of sequence conservation between cultivar R570 and *S. officinarum*, with 215 genes showing 100% identity, 134 genes at 99%, and 114 genes at 98% identity (**Supplementary Figure S6**). This suggests a prominent genomic relationship with *S. officinarum*, while also highlighting significant variation compared to *S. spontaneum*.

To ensure a unified nomenclature for easy comparison, each gene was renamed as SoffiXPP2C, followed by a unique number assigned based on its chromosomal position. This systematic naming approach facilitates both intra and interspecies comparisons, assisting in the functional characterization of homologs and the identification of orthologous relationships with model plant species. However, further investigation is needed to determine whether biological functions are consistently conserved within these relationships (Hamel et al., 2014; Kumari et al., 2022).

The physiological properties of PP2C proteins, including gene and protein length (amino acids), molecular weight, and isoelectric point, varied widely. Additionally, subcellular localization predictions indicated that the identified PP2C proteins were distributed across different cellular compartments, indicating their diverse functional roles (**Supplementary Table S1**). A total of ten motifs were identified in the SoffiXPP2C genes (**Supplementary Figure S2**). Specifically, motifs 1, 3, 4, and 6 were consistently present in all analyzed genes, indicating that these motifs are likely involved in representing PP2C domains. Proteins within the same group displayed similar motif distribution patterns. Certain motifs were uniquely present in specific subfamilies, whereas others are commonly found across most subfamilies. This distribution pattern suggests a potential functional divergence among the various subfamilies. All PP2C members were found to contain the PP2C domain, as identified through the CDD program. The localization of the PP2C domain was highly conserved across different PP2C subfamilies, indicating a strong structural and functional conservation within this gene family. Gene structure analysis of the PP2C family across different plants revealed a certain degree of structural conservation (**Supplementary Figure S1**). The highest exon count varied among species, with *S. spontaneum* having up to 14 exons, Arabidopsis having maximum of 12 exons, rice with 18 exons, and *Brassica rapa* with 19 exons. In SoffiXPP2C, the highest exon count was 20 (Khan et al., 2019; Xue et al., 2008; Huang et al., 2023). Additionaly, exon distribution within members of the same subfamily was often consistent. This result indicates a close evolutionary relationship and conserved gene structure across different plant species. Functional analysis using the InterPro database revealed significant structural diversity among sugarcane PP2C phosphatases, emphasizing their potential roles in stress signaling, protein regulation, and cellular adaptation. The presence of multiple conserved domains, including PP2C catalytic domains, protein kinase domains, and other functional motifs, suggests a broad functional spectrum of the PP2C gene family in sugarcane. This highlights the need for further functional validation to determine their precise contributions to abiotic stress tolerance.

**Figure 9:**
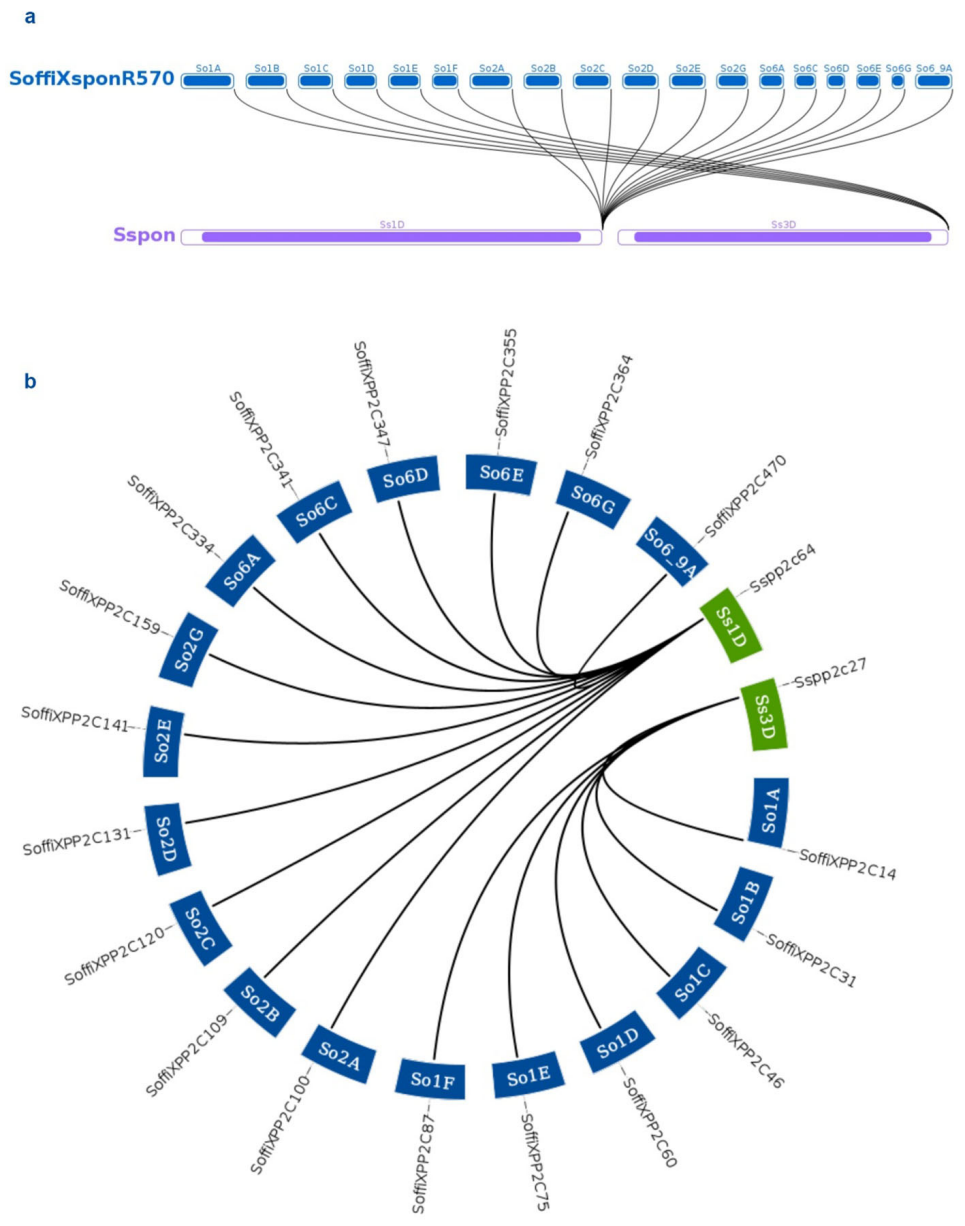
Synteny relationships depicted using **(a)** a linear synteny plot and **(b)** a Circos plot to illustrate the genomic association between two potential cold stress-responsive genes, *SsPP2C27* and *SsPP2C64*, from the PP2C gene family in *Saccharum spontaneum*, and their homologs from the SoffiXPP2C gene set. SoffiXPP2C stands for “*Saccharum officinarum* and *Saccharum spontaneum hybrid”* and PP2C represents “Protein Phosphatase 2C; Sspp2c: *Saccharum spontaneum* PP2C.

The distribution of 500 PP2C genes across all 10 chromosomes and their subgenomes (**Figure 2**), along with their presence in interspecific recombinant regions and scaffolds, highlights the complex genomic organization and evolutionary expansion of the PP2C family in sugarcane. Among the 500 PP2C genes, 297 were located on chromosomes 1 to 5, indicating a biased distribution pattern across the genome. Synteny analysis was performed between the sugarcane hybrid genome and the *S. spontaneum* genome to understand the conservation of the genomic context of PP2C genes between the two species (**Figure 3**). The synteny analysis revealed a total of 62 synteny networks involving 477 out of 500 SoffiXPP2C genes and 156 genes (76 PP2C genes and 80 other genes) from *S. spontaneum*. Notably, only the PP2C genes identified in the sugarcane genome in current study were exhibited synteny associations, indicating strong evolutionary conservation and functional significance within this gene family. In the study on *S. spontaneum*, 27 SsPP2C genes exhibited cold-stress-induced expression, with *SsPP2C27* and *SsPP2C64* identified as potential hub genes involved in ABA signal transduction, suggesting their key roles in stress adaptation (Huang et al., 2023). In the synteny analysis, 13 out of these 27 SsPP2C genes were found to show synteny association. Among them, *SsPP2C27* and *SsPP2C64* were also involves in synteny relationships within the same network (**Figure 9**). This indicates a possible role of SoffiXPP2C genes in cold stress adaptation.

From that synteny network, 4 genes *SoffiXPP2C31, SoffiXPP2C75, SoffiXPP2C60*, and *SoffiXPP2C14* have been studied for genomic DNA amplification in 5 sugarcane cultivar and cDNA amplification from waterlogging stressed root and stem sample from sugarcane cultivar. Additionally, RT-qPCR study has been done to evaluate gene expression pattern of these four gene in waterlogging stressed samples from 5 sugarcane cultivar. The successful amplification of all four SoffiXPP2C genes across sugarcane cultivars in genomic and waterlogging stress cDNA sample confirms their presence and suggests they may play important roles in plant responses to waterlogging stress. Differential expression patterns observed in stem and root tissues under waterlogging stress indicate possible gene-specific functions in stress adaptation mechanisms.

The downregulation of some of the SoffiXPP2C genes in tolerant genotypes showed that negative regulator role of these genes in particular for waterlogging stress. PP2C phosphatases are well-documented negative regulators of ABA signaling (Xing et al., 2021). For example, wheat (TaPP2C59) and Maize (ZmPP2C) were shown as negative regulator of salt and drought stress responses (Guo et al., 2023; Liu et al., 2009). The differential regulation of these genes under stress conditions indicates that they may be involved in fine-tuning the plant’s signaling networks, potentially offering cross-protection against multiple environmental challenges. It was demonstrated that under hypoxia stress, the expression level of the *Arabidopsis* PP2Ac gene was reduced in whole 7-day-old seedlings after 2 and 9 hours of treatment compared to control plants (Branco-Price et al., 2008). When the stress is relieved, the PP2Ac transcript level increased relative to hypoxia-treated plants. In contrast, the transcript level of PP2Ac remained similar in *Arabidopsis* plants grown under ambient air in darkness and those subjected to full submergence in darkness for 4 hours (Van Veen et al., 2016).

An analysis of the structure domain of all *S. spontaneum* genes involved in synteny revealed the presence of multiple conserved domains, including PP2C catalytic domains, protein kinase domains, and other functional motifs, suggesting a broad functional spectrum of the PP2C gene family in these gene. Essential PP2C domains were consistently present across most gene members, indicating their crucial role in maintaining phosphatase activity and regulatory functions. The presence of multiple functional motifs and interaction domains in SoffiXPP2C and the genes associated with it in synteny suggests that the identified PP2C proteins in sugarcane may play critical roles in stress signaling, protein phosphorylation regulation, and other cellular regulatory mechanisms.

Gene duplication is a pivotal mechanism in evolution, significantly contributing to genetic diversity and the adaptation of organisms. It can occur through various processes, such as unequal crossing over and whole-genome duplications, leading to the emergence of new gene functions and increased genomic plasticity (Magadum et al., 2013). The present study identified 107 duplicated SoffiXPP2C genes out of 500, exhibiting tandem duplication, indicating the presence of paralogous gene pairs in the sugarcane genome (**Figure 4**). Interestingly, previous studies on gene duplication have shown a predominance of segmental duplications over tandem duplications in other species. For instance, in sunflower, only five out of 53 PP2C gene pairs exhibited tandem duplications, while 48 pairs showed segmental duplication. Similarly, in rice, there were 12 segmental and 4 tandem duplicated gene clusters (Xue et al., 2008). Notably, no tandem duplications have been reported in the PP2C gene families of *Brachypodium distachyon*, *Medicago truncatula*, soybean (*Glycine max*), *Cucumis sativus* L and, *Arachis hypogaea* (Cao et al., 2016; Yang et al., 2018; Fan et al., 2020; Zhang et al., 2022; Wu Z. et al., 2023). This observation contrasts with the findings of the present study, where SoffiXPP2C genes predominantly exhibit tandem duplication rather than segmental duplication, suggesting a unique evolutionary pattern in the expansion of the sugarcane PP2C gene family. Tandem duplications can lead to sub-functionalization, where duplicated genes may acquire distinct roles, enhancing the adaptability of plants to various stresses (Fan et al., 2020; Lallemand et al., 2020). Pairwise synonymous (Ks) and non-synonymous (Ka) substitution rate analysis revealed (**Supplementary Table S8**) that most gene pairs had a Ka value of 0. Only two pairs (e.g., *SoffiXPP2C82–SoffiXPP2C54* and *SoffiXPP2C89–SoffiXPP2C49*) showed Ka/Ks ratios below 1, indicating purifying or stabilizing selection. Similar analyses in previously studied crops such as Brassica, sunflower, soybean, and peanut have also reported Ka/Ks ratios below 1 (Akter et al., 2024; Khan et al., 2019; Fan et al., 2020; Wu et al., 2023). Most of the gene pairs exhibited low Ks values (< 0.05), indicating that these duplication events are relatively recent (Lu et al., 2012; Bu and Katju, 2015).

The identification of 34 PP2C genes aligning with five differentially expressed contigs in the sugarcane hybrid cultivar provides valuable insights into the potential role of PP2C phosphatases in stress response. Functional annotation from UniProt further supported the involvement of these contigs in stress response pathways, as most were associated with protein-serine/threonine phosphatases, PPM-type phosphatase domain-containing proteins, and RNA-processing functions. The identification of one contig as glycerol kinase also suggests a potential link between stress response and metabolic regulation. These findings underscore the significance of PP2C genes in sugarcane’s adaptation to environmental stressors and highlight the need for further functional validation to elucidate their precise regulatory mechanisms (Petelenz-Kurdziel et al., 2013; Park et al., 2017).

The GO terms analysis indicates significant roles of SoffiXPP2C genes in responding to both biotic and abiotic stressor responses and key metabolic pathways. Enriched terms include protein serine/threonine phosphatase activity, which is vital for regulating various cellular processes (Mumby and Walter, 1993; Brautigan and Shenolikar, 2018). Genes associated with cellular components like nucleus, cytosol and plasma membrane indicate their involvement in essential cellular functions, such as gene expression regulation, signal transduction, and maintaining cellular integrity (Verstraeten et al., 2007).

This study identified 500 SoffiXPP2C genes in sugarcane, highlighting their conserved domains, functional diversity, and roles in abiotic stress response and gene family expansion. It also highlights their syntenic conservation with *S. spontaneum* and the expression pattern of SoffiXPP2C have shown its role in waterlogging stressed condition. These findings provide valuable insights into PP2C phosphatases in sugarcane, laying the foundation for future research on stress response mechanisms and the development of stress-tolerant cultivars.

## Data Availability

Publicly available datasets were analyzed in this study. This data can be found here: Not applicable.
