# Supplementary material for: Comparative genomics and synteny analysis of PP2C phosphatases in modern and wild sugarcane cultivars for insights into abiotic stress response
Source: Front Plant Sci. 2025 Aug 19;16:1596800. doi: 10.3389/fpls.2025.1596800 (PMC12401961; doi:10.3389/fpls.2025.1596800)
Supplement: Supplementary file 1 [file DataSheet1.docx]

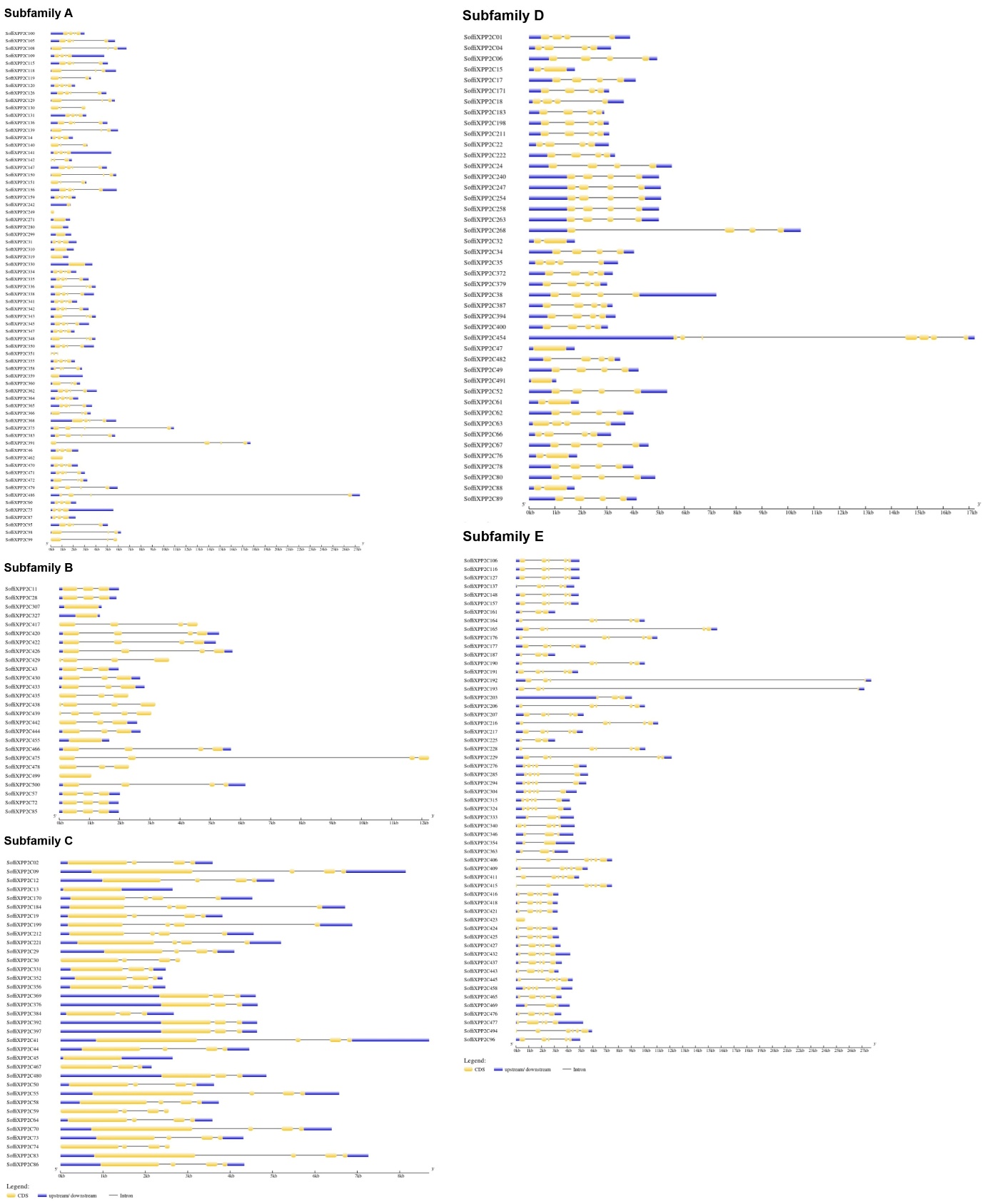


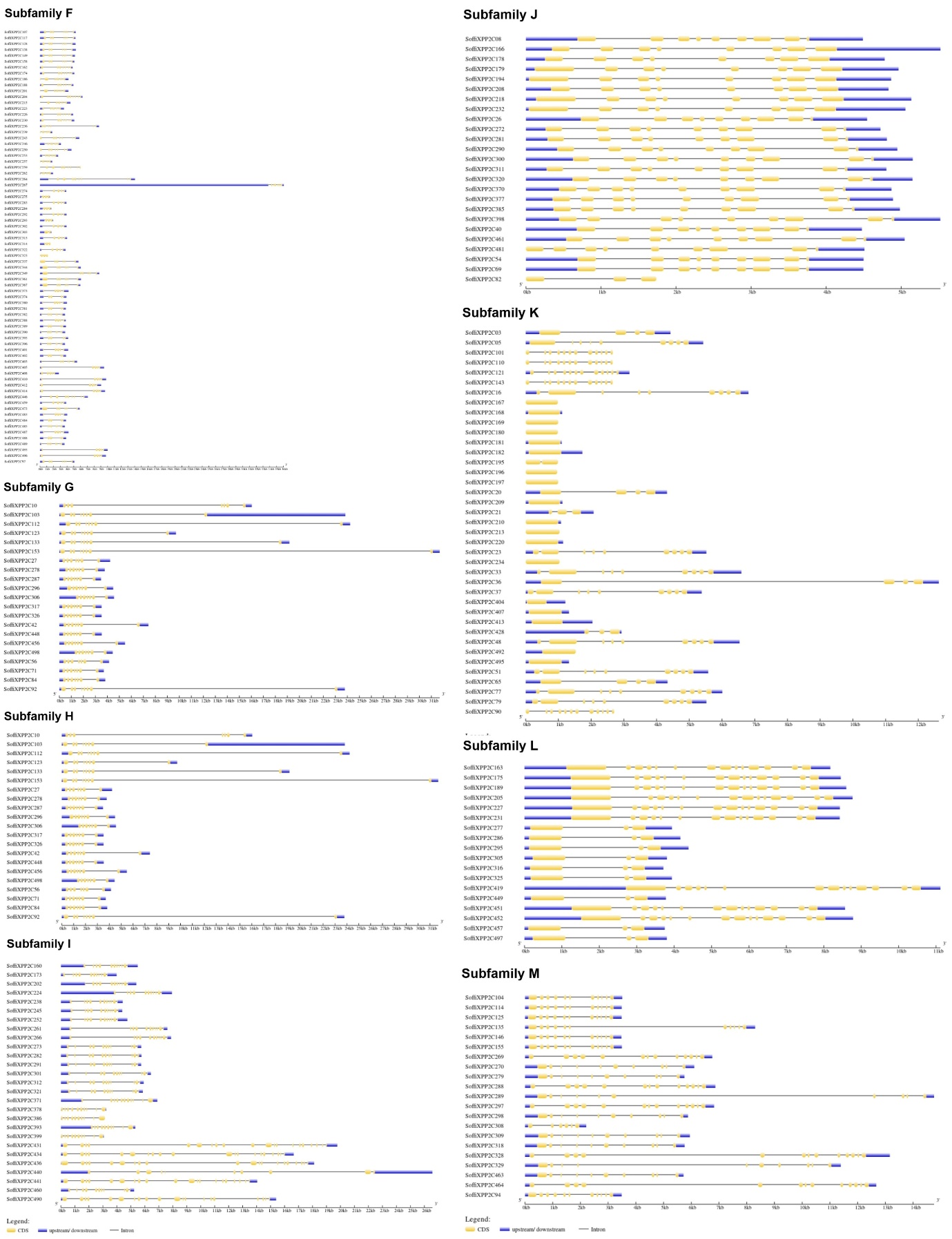


**Figure S1|** Gene structure of SoffiXPP2C genes. The Yellow bars represent exons, the black line between them represents introns and blue bar represents untranslated region. The scale at the bottom indicates the length of gene


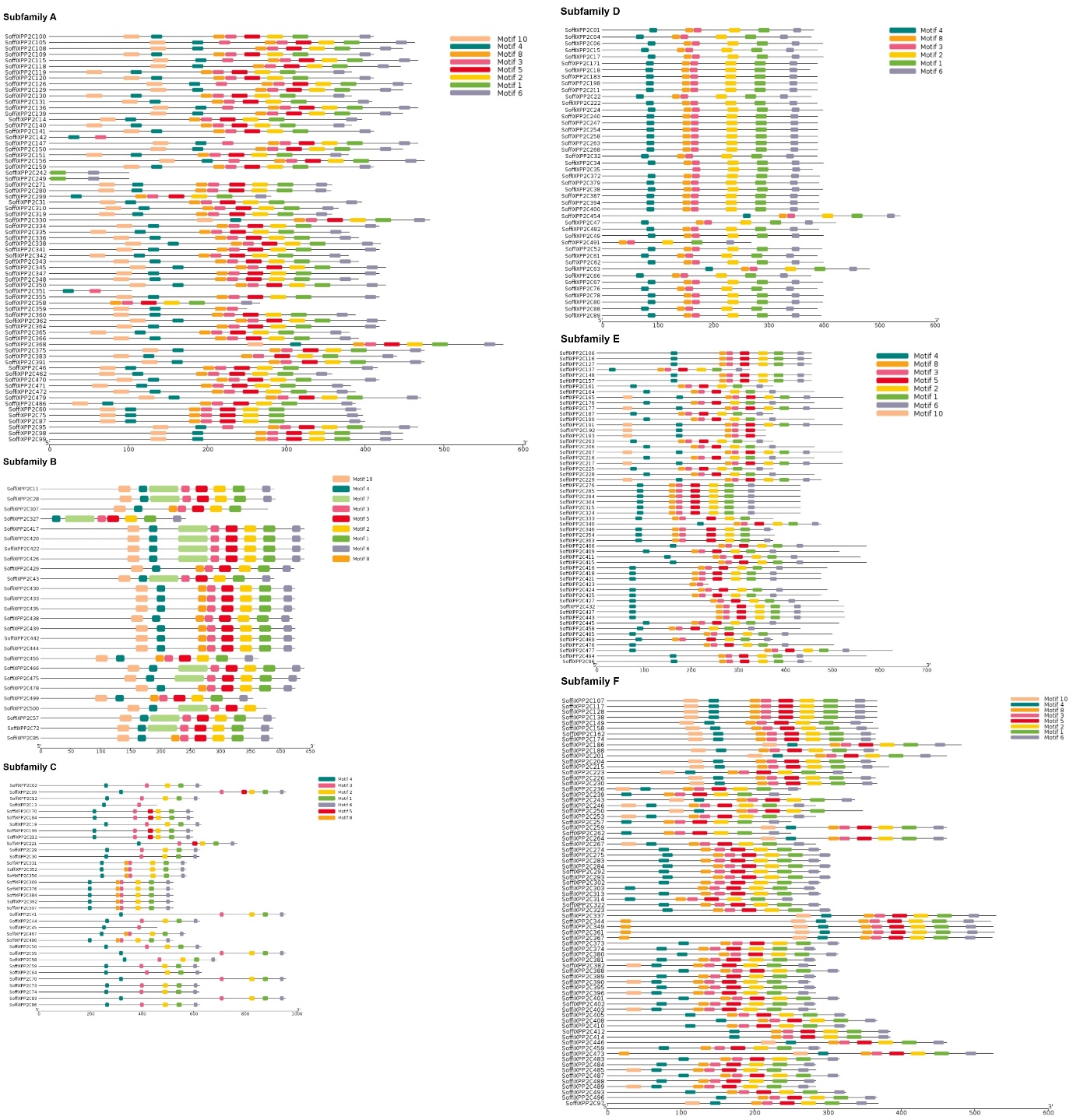


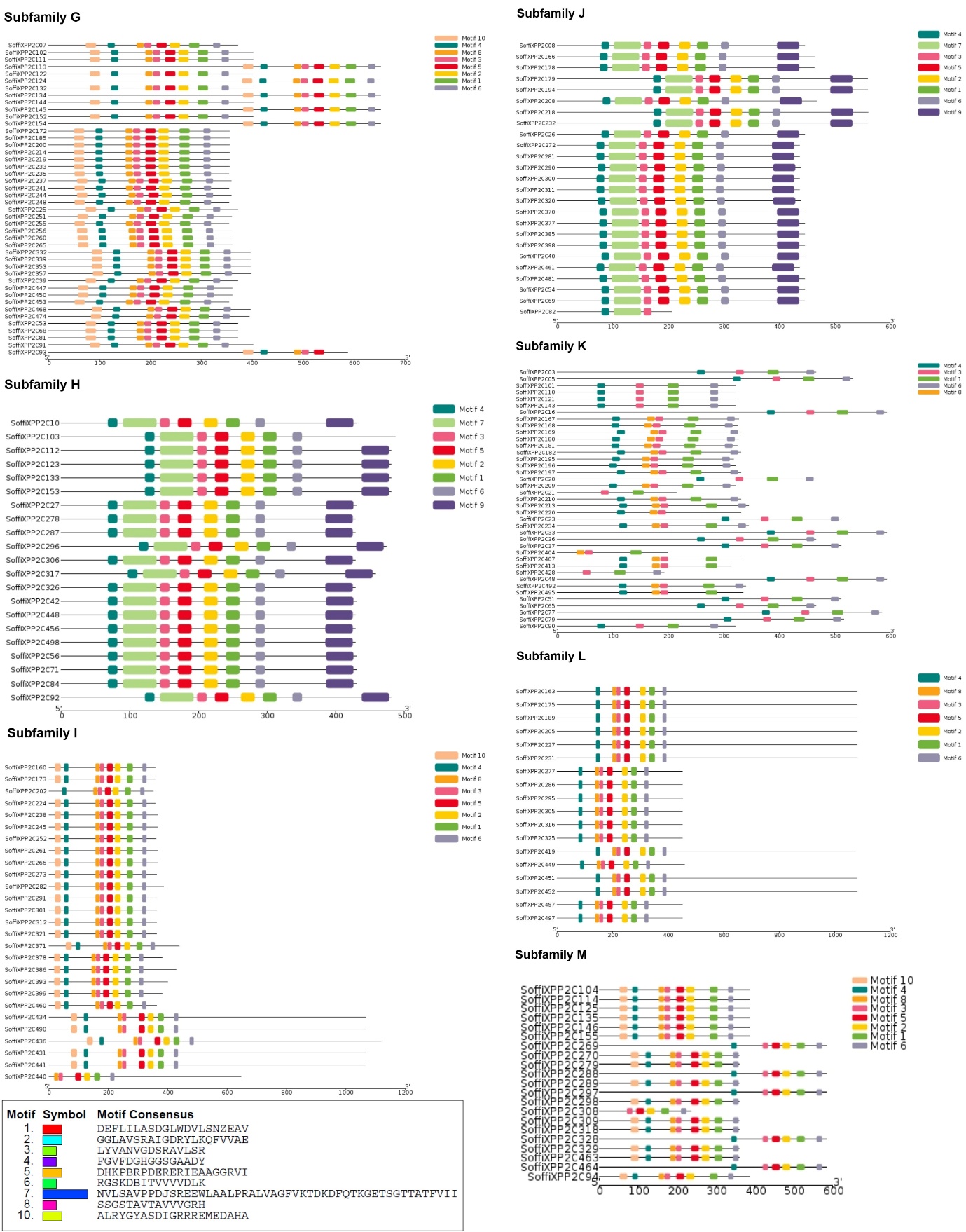


**Figure S2|** Conserved motif in SoffiXPP2C genes. The different color box represents the different 10 motifs.


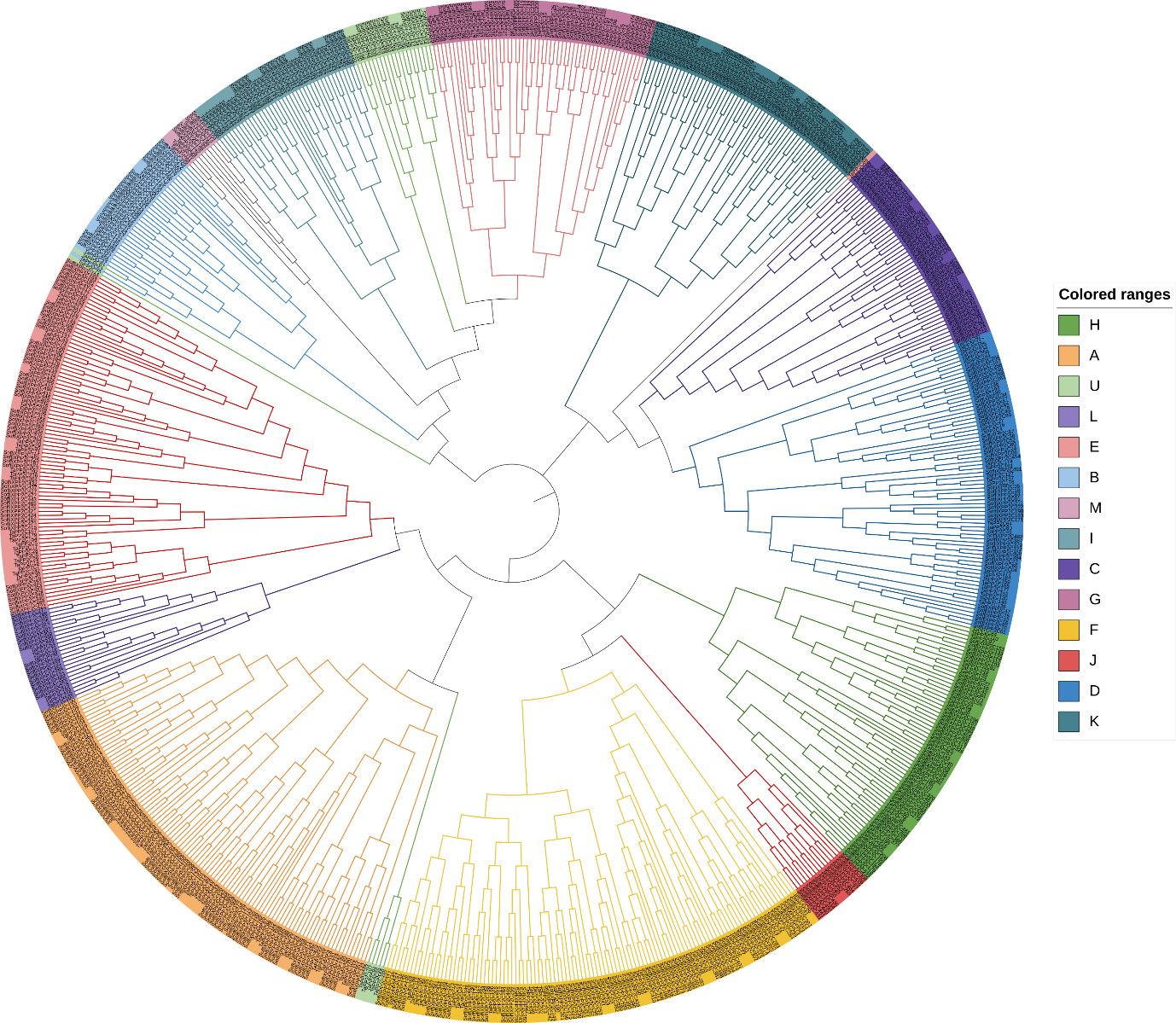


**Figure S3|** Phylogenetic relationship among the 500 SoffiXPP2C genes and PP2C genes of wheat.


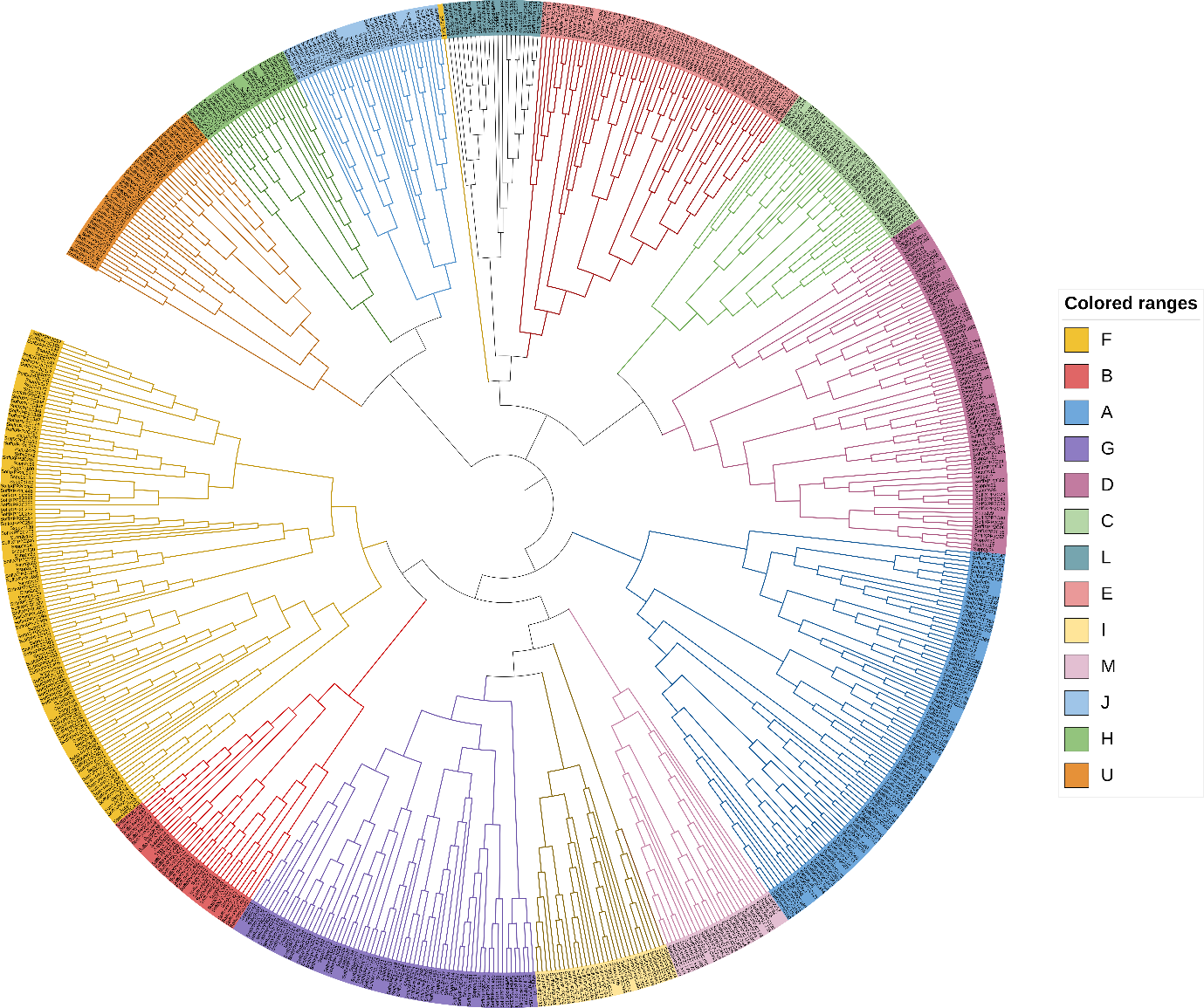


**Figure S4|** Phylogenetic relationship among the 500 SoffiXPP2C genes and PP2C genes of wild sugarcane (*Saccharum spontaneum)*.


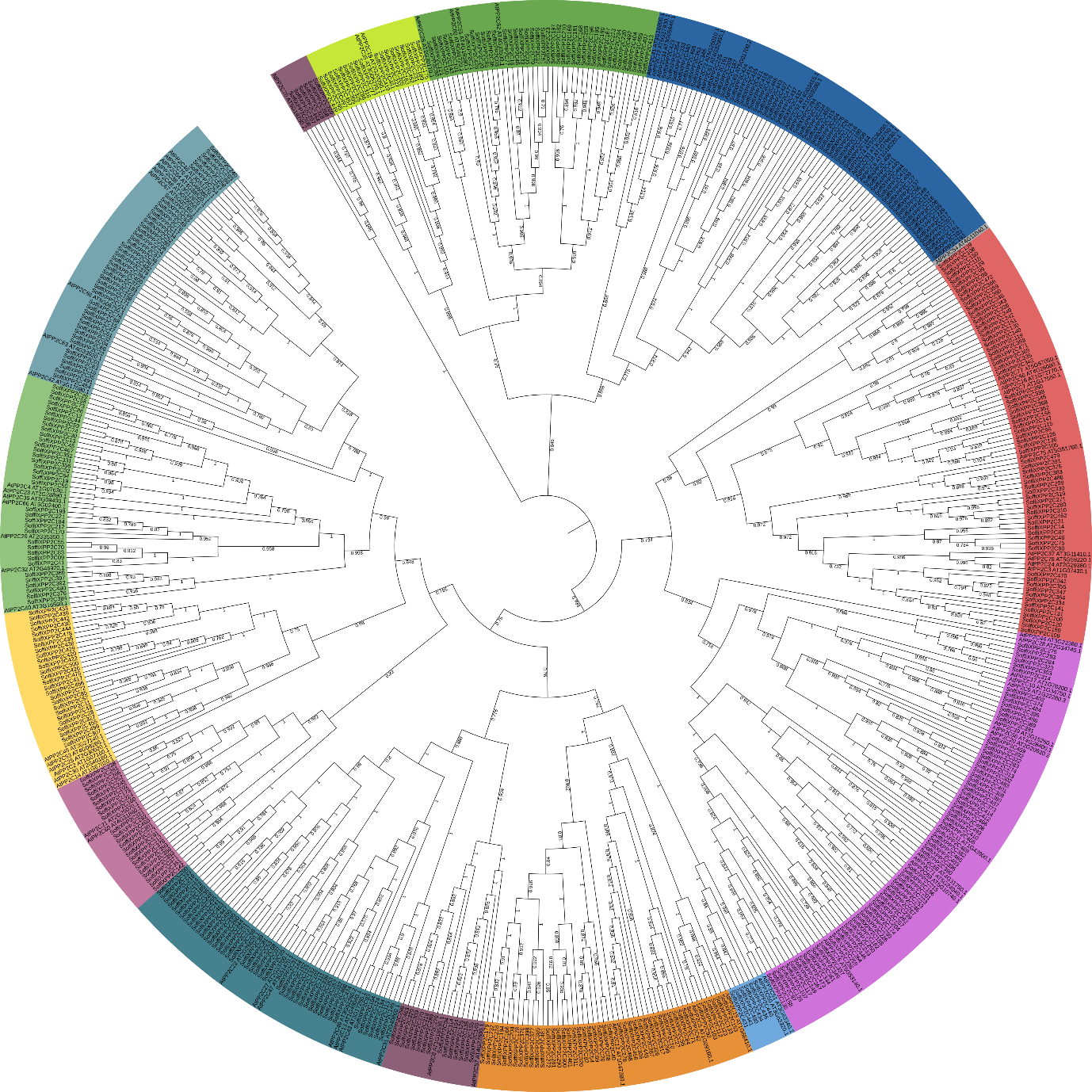


**Figure S5|** Phylogenetic relationship among the 500 SoffiXPP2C genes and PP2C genes of *Arabidopsis thaliana*.


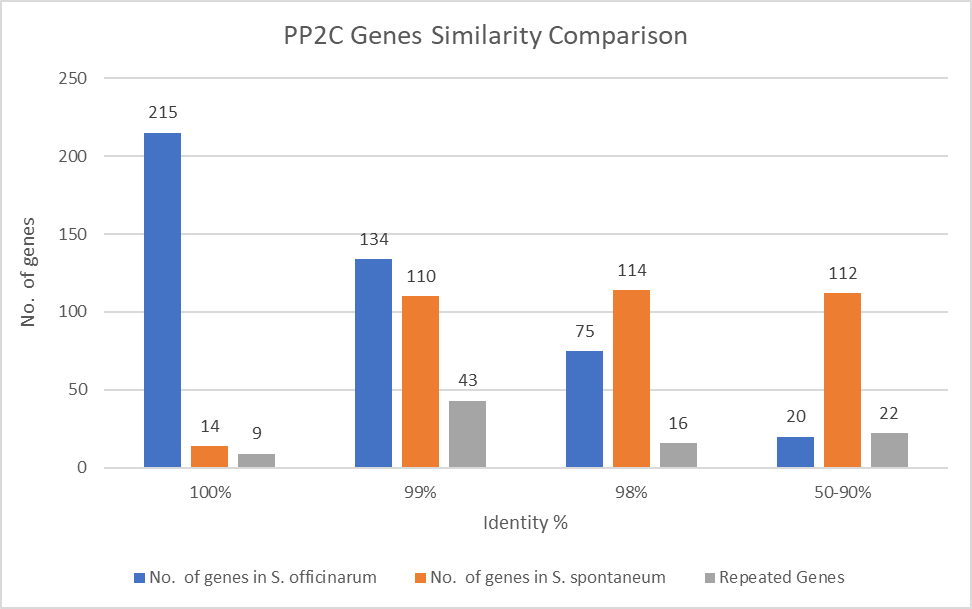


**Figure S6|** Comparison of PP2C gene similarity levels between Sugarcane cultivar R570 and its wild ancestors, *S. officinarum* and *S. spontaneum*. The bar chart shows the number of PP2C genes exhibiting different levels of sequence identity (100%, 99%, 98%, and 50-90%) with each wild ancestor. The “Repeated Genes” category represents genes shared between both wild species at each similarity level.
